# Exploiting the MDM2-CK1α Protein-Protein Interface to Develop Novel *Biologics* That Induce UBL-Kinase-Modification and Inhibit Cell Growth

**DOI:** 10.1371/journal.pone.0043391

**Published:** 2012-08-20

**Authors:** Anne-Sophie Huart, Nicola J. MacLaine, Vikram Narayan, Ted R. Hupp

**Affiliations:** 1 p53 Signal Transduction Group, Edinburgh Cancer Research Centre in the Institute of Genetics and Molecular Medicine, University of Edinburgh, Edinburgh, United Kingdom; 2 Interferon and Cell Signalling Group, Edinburgh Cancer Research Centre in the Institute of Genetics and Molecular Medicine, University of Edinburgh, Edinburgh, United Kingdom; Spanish National Cancer Center, Spain

## Abstract

Protein-protein interactions forming dominant signalling events are providing ever-growing platforms for the development of novel *Biologic* tools for controlling cell growth. Casein Kinase 1 α (CK1α) forms a genetic and physical interaction with the murine double minute chromosome 2 (MDM2) oncoprotein resulting in degradation of the p53 tumour suppressor. Pharmacological inhibition of CK1 increases p53 protein level and induces cell death, whilst small interfering RNA-mediated depletion of CK1α stabilizes p53 and induces growth arrest. We mapped the dominant protein-protein interface that stabilizes the MDM2 and CK1α complex in order to determine whether a peptide derived from the core CK1α-MDM2 interface form novel *Biologics* that can be used to probe the contribution of the CK1-MDM2 protein-protein interaction to p53 activation and cell viability. Overlapping peptides derived from CK1α were screened for dominant MDM2 binding sites using (i) ELISA with recombinant MDM2; (ii) cell lysate pull-down towards endogenous MDM2; (iii) MDM2-CK1α complex-based competition ELISA; and (iv) MDM2-mediated ubiquitination. One dominant peptide, peptide 35 was bioactive in all four assays and its transfection induced cell death/growth arrest in a p53-independent manner. Ectopic expression of flag-tagged peptide 35 induced a novel ubiquitin and NEDD8 modification of CK1α, providing one of the first examples whereby NEDDylation of a protein kinase can be induced. These data identify an MDM2 binding motif in CK1α which when isolated as a small peptide can (i) function as a dominant negative inhibitor of the CK1α-MDM2 interface, (ii) be used as a tool to study NEDDylation of CK1α, and (iii) reduce cell growth. Further, this approach provides a technological blueprint, complementing siRNA and chemical biology approaches, by exploiting protein-protein interactions in order to develop *Biologics* to manipulate novel types of signalling pathways such as cross-talk between NEDDylation, protein kinase signalling, and cell survival.

## Introduction

CK1 human isoforms - α, γ1, γ2, γ3, δ and ε - represent a unique group within the superfamily of serine/threonine specific protein kinases that function as monomeric and constitutively active enzymes [1], [2]. They differ significantly in the length and primary structure of their C-terminal non-catalytic domain which is an extended tail in the case of δ/ε as opposed to α which has a limited C-terminal domain, but CK1γ isoforms on the other hand vary in a longer N-terminal head [3]. Although CK1 isoforms and associated splice variants are ubiquitously expressed, their activity is greatly regulated via their expression levels [4], post-translational modifications by various mechanisms including subcellular stimuli [5], [6], subcellular compartmentalisation [7], [8], proteolytic cleavage of the C-terminus, auto- and de-phosphorylation of the C-terminal regulatory domain [9]. CK1s, which were among the first kinases described and were named after the use of casein in the assessment of their kinase activity, have been involved in a plethora of pathways responsible for differentiation [10], proliferation/cell cycle progression [11], chromosome segregation [12], membrane trafficking [13], [14], circadian rhythms [15], apoptosis [16], translation initiation [17], and cell migration [18], [19]. Therefore CK1 deregulation has been linked to neurodegenerative diseases like Alzheimer’s, sleeping disorders and proliferative diseases such as cancer. Several CK1 specific inhibitors have been described, among them D4476 (4[4-(2,3-dihydro-benzo[1], [4]dioxin-6-yl)-5-pyridin-2-yl-1-H-imidazol-2-yl]benzamidine) which is an ATP-competitive inhibitor active on CK1 in the nanomolar range *in vitro* [20], [21]. In addition, a novel stimulator of CK1α has been identified named pyrvinium that opens the door to pharmacological induction of CK1α flux in cells [22], [23].

The transcription factor p53 is a tumour suppressor protein that prevents propagation of damaged cells with potentially cancer-prone mutations by triggering cell cycle arrest, repair mechanisms, apoptosis, and immune system engagement [24], [25], [26]. Mutational inactivation of p53 leads to genome instability, immune system evasion, and metabolic stress in human tumours that can mediate drug resistance. Nonetheless, some p53 mutant tumours can allow sensitization to specific therapeutic interventions [27]. Under normal conditions, p53 is negatively regulated by the murine double minute chromosome 2 (MDM2) protein which results in both ubiquitination and subsequent degradation of p53 by the proteasome as well as direct repression of p53 transcription activity [28]. The mechanism is controlled through the ability of p53 to transcriptionally activate the *mdm2* gene in a negative feedback loop [29], [30]. MDM2 has been divided into several domains [31]: a regulatory lid; an N-terminal allosteric hydrophobic pocket; a nuclear localization signal and a nuclear export signal; an intrinsically disordered acidic domain that drives a large number of MDM2 interactions; a C-terminal RING domain; and an ATP-binding motif. Ubiquitin ligase function of MDM2 toward p53 has been shown to involve a two-site docking model: occupation of the N-terminal hydrophobic pocket of MDM2 by a motif within the N-terminus of p53 induces docking between the acidic domain of MDM2 and an ubiquitin-signal in the DNA-binding domain of p53 [31].

The dynamic interaction between p53 and MDM2 relies on integration of post-translational modifications driven by multiple signalling pathways [26], [32]. Phosphorylation of both p53 and MDM2 can be regulated by the same kinase, Casein Kinase 1 (CK1) isoforms - α, δ, and ε - which has been shown to phosphorylate p53 after transforming growth factor beta [33], some DNA damage signals [34] or virus infection [6], which frees p53 from MDM2 [35]. CK1 has also been shown, in proliferating conditions, to phosphorylate residues within the acidic domain of MDM2, favouring MDM2 functions toward p53 [36], [37], [38]. CK1 would thus have a dual role, possessing some proto-oncogene functions on one hand but it could switch towards a tumour suppressor function, depending on recruitment into specific complexes under different conditions.

Disruption of the p53-MDM2 complex using chemicals like Nutlin-3 that destabilise the protein-protein interface between MDM2 and p53 is a fundamental pharmacologic approach for p53 activation as a cancer therapeutic. Our previous data indicated that CK1 and MDM2 form a stable protein-protein complex that regulates the steady-state levels of the p53 tumour suppressor protein in cancer cell lines [16]. The observation that a small molecule inhibitor of CK1 can act like the MDM2 binding molecule Nutlin to stabilize p53 and induce cell death, also suggests that CK1 forms an attractive target for anti-cancer therapeutics. In this study, we aimed to determine whether disruption of the protein-protein interaction between MDM2 and CK1α give rise to similar and/or novel biological insights compared to the use of siRNA or small molecule pharmacological kinase inhibitors that either deplete CK1 protein or “inhibit” kinase activity but do not lead to protein kinase degradation, respectively. This concept is relatively important to begin to address since there is growing interest in “drugging” protein-protein interactions as therapeutic strategies in disease like cancer [39], [40]. We define a dominant functional motif in CK1α that forms the core protein-protein interface between MDM2 and CK1α using a functional *in vitro/in vivo* peptide-binding screen. This research led to the identification of a specific peptide derived from the “base” of CK1α capable of de-stabilizing the MDM2-CK1α complex, and inducing p53-independent cell death. This approach highlights how protein-protein interfaces can be exploited to develop novel *Biologics*, which can be used to dissect signal transduction mechanisms and provide leads for novel therapeutics to target specific MDM2 protein-protein interactions in cancer cells [41].

## Results

### CK1α Protein Depletion or Pharmacological Kinase Inhibition Activates p53 and Induces its Nuclear Relocalisation Revealing a CK1α-MDM2 Complex

**Figure 1:**
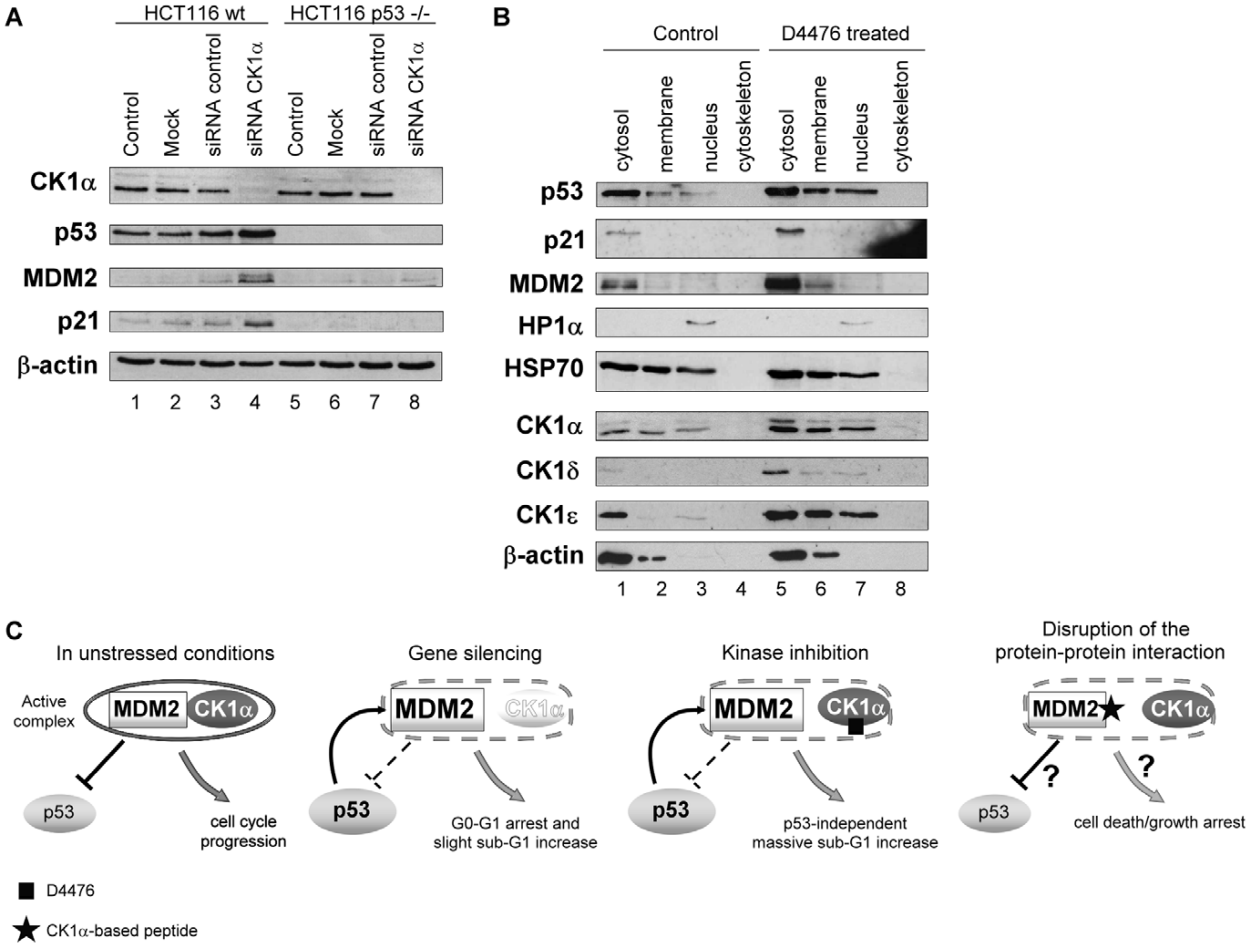
Effects of CK1α depletion or inhibition on the stability and subcellular localisation of the p53 protein. (**A**) HCT116 wt (lanes 1–4) and p53−/− (lanes 5–8) cells were transfected with control (40 nM; lanes 3&7) or with CK1α-specific siRNA (40 nM; lanes 4&8) for 96 hours. A Mock transfected control (lanes 2&6) and an untreated control (DMEM only; lanes 1&5) were included. Cell lysates were analysed by Western blotting with antibodies targeting the indicated proteins. (**B**) A375 cells were transfected with 40 µM final concentration of the CK1 inhibitor D4476 for 72 hours (lanes 5–8). A Mock/DMSO control (lanes 1–4) was included. Cells were fractionated into four subcellular compartments: cytosol, membranes and membrane organelles, nucleic proteins, and cytoskeletal components. Subcellular fractions were immunoblotted with antibodies targeting the indicated proteins. (**C**) Strategies for manipulating signalling complexes under normal growing conditions, CK1α interacts with MDM2, which promotes its binding to p53 and leads to p53 ubiquitination and degradation but also to inhibition of transactivation of p53 targets, p21 for example. After genetic depletion of CK1α by siRNA, the loss of the bioactive complex between MDM2 and CK1α could explain the stabilization of p53. Alternatively, the MDM2-CK1α complex might be inactivated for example by using an ATP-competitive inhibitor D4476 which induced p53 accumulation and cell death. The approach developed here was to target the dominant sit of protein-protein interaction between the MDM2-CK1α complex to identify a potentially bioactive CK1α interface peptide that would form a new specific *biologic* tool to manipulate cancer cell growth.

CK1α was recently identified as a repressor of the p53 pathway in A375 cells; siRNA against CK1α or D4476, a specific ATP-competitive kinase inhibitor against CK1, resulted in accumulation of the p53 protein [16], as previously reported after IC261 treatment [42]. These results were reproduced in HCT116 colorectal cancer cells (Figure 1A) and also in A375 cancer cells (Figure S1) with isoform-specific siRNA against *CK1α* (*CSNK1A1* gene), which reduced specifically the levels of CK1α (Figure 1A lanes 4&8 vs. 3? Figure S1). In p53 wt cells (Figure 1A lanes 1–4), the down-regulation of CK1α protein triggered p53, MDM2 and p21 increases. The proposed mechanism for these changes in p53/MDM2 pathway is a disruption between MDM2 and p53 binding after CK1α depletion, leading to an increase of p53 protein along with an increase of p53-inducible gene products MDM2 and p21. Indeed these observations matched the effects of Nutlin-3a treatment [16] and are consistent with the ability of CK1 to induce MDM2 phosphorylation within its acidic domain, which is believed to positively control MDM2 activities toward p53 [38]. Nuclear localization is critical for wild type p53 transactivation function. Here, we examined the effect of CK1 inhibition on the subcellular localization of p53 in A375 cell line in which p53 is normally mainly sequestered in the cytoplasm in unstressed conditions (Figure 1B lanes 1–4). D4476 treatment triggered an increase of nuclear p53 protein level (Figure 1B lanes 3 vs. 7; Figure S2), while a slighter increase in cytosolic p53 protein level was observed (Figure 1B lanes 5 vs. 1; Figure S2).

Expression of CK1 isoforms were also assessed and interestingly after D4476 treatment, CK1α, δ and ε protein levels increased significantly (Figure 1B), which highlights the different effects that siRNA or a small molecule pharmacological inhibitor can have on target protein levels in cells. That is, it cannot be assumed that kinase inhibition is genetically identical to removing a protein using siRNA or gene knockout approaches. As a control, CK1δ and ε levels didn’t increase after treating cells with CK1α targeted siRNA (Figure S1). Such increases of CK1δ and ε were previously observed after IC261 treatment [42], another ATP-binding pocket CK1 inhibitor [43]. These increases were explained as a response to compensate the inhibition of casein kinase 1 isoforms [44]. After D4476 treatment, CK1α protein cytosolic level increased by about 3-fold, δ by 6-fold and ε by 2-fold (Figure S2), which could reflect the different inhibition sensitivity of the distinct isoforms by D4476. But it was previously shown in A375 cells that p53, MDM2 and p21 protein level changes couldn’t be attributed to other isoforms as CK1δ siRNA for example didn’t induce these protein level increases [16].

Two standard approaches are used to dissect a signalling pathway; either targeting components at the mRNA level with specific siRNA or at the protein level with small molecule inhibitors. There is a third way to manipulate signalling complex like CK1α-MDM2, by targeting the interface to discover a new *biologic* tool whose effects on cell death/growth arrest abilities can be investigated (Figure 1C). Targeting protein-protein interactions is growing as a dominant approach in therapeutics and in this manuscript we develop a biologic tool to this interface to compare siRNA and small molecule inhibitors in order to develop new insights into signalling and novel therapeutic approaches for targeting MDM2 and CK1 in cancer (Figure 1C).

### CK1α Transcript Variant 2 Forms a Stable Multi-protein Complex with MDM2 in Cells

**Figure 2:**
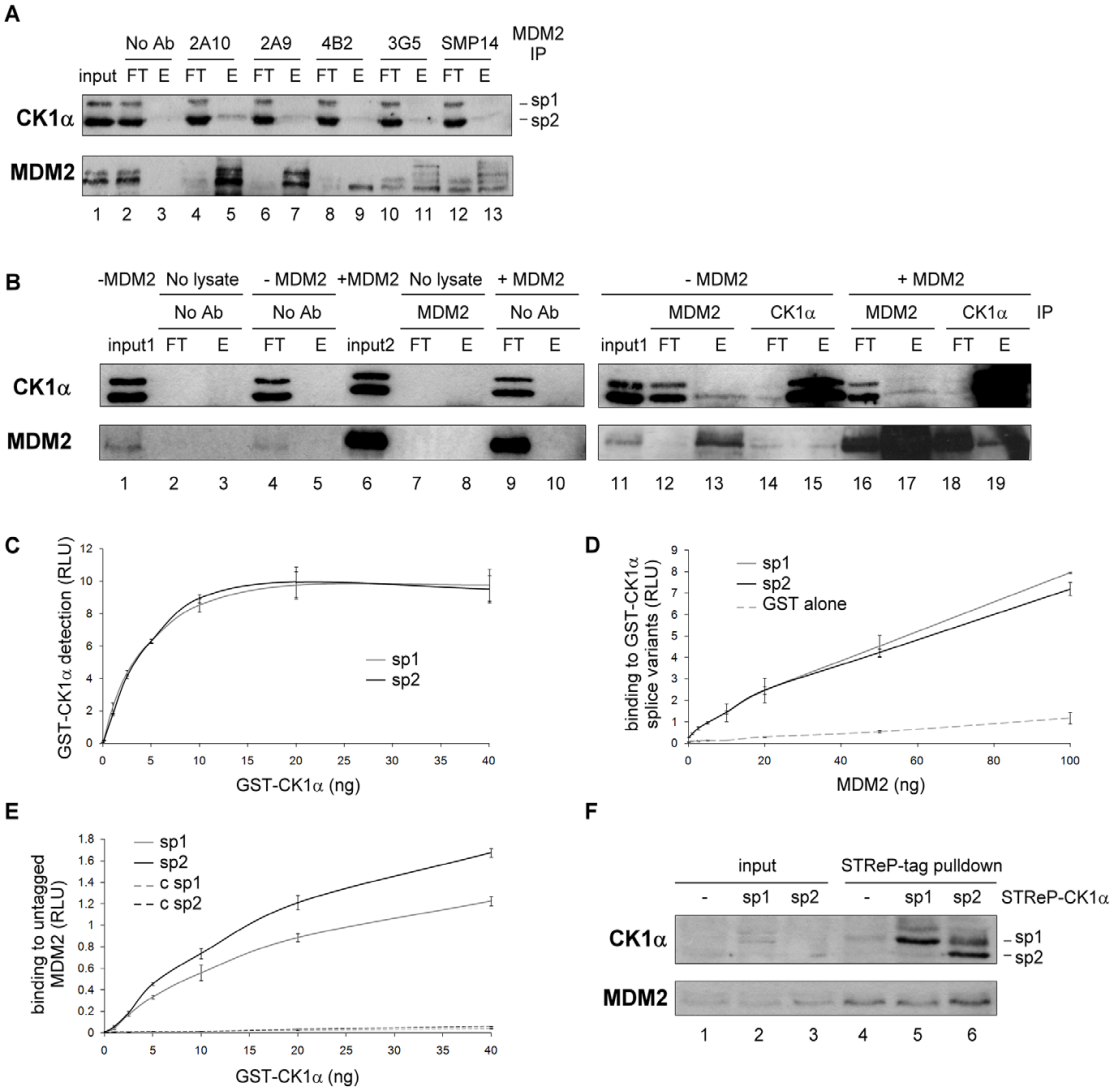
MDM2 preferentially binds CK1α splice variant 2. (**A**) MDM2 was immunoprecipitated from A375 cell lysate using a panel of MDM2 antibodies: 2A10, 2A9, 4B2, 3G5 and SMP14. Co-immunoprecipitation was performed and proteins in the immune precipitate were analysed by Western blotting with anti-CK1α and antibody targeting the immunoprecipitated MDM2 protein (1∶1 mix of 2A10 and 4B2). A “no antibody” control (No Ab) was included. The flow-through (FT) and the eluate (E) were analysed along with the pre-cleared lysate (input). (**B**) MDM2 or CK1α was immunoprecipitated from cell lysates transfected (lanes 16–19) or not (lanes 11–15) with untagged MDM2. Co-immunoprecipitation was performed and proteins in the immune precipitate were quantified by Western blotting with anti-CK1α and anti-MDM2 4B2 antibodies. Various immunoprecipitation controls were included (lane 2–5 and 7–10) and inputs for each lysate (lanes 1 & 6; input 1: non-transfected cells, input 2: MDM2 transfected cells) (**C**) Normalisation of GST-CK1α splice variant 1 and 2 proteins (used in (D) and (E)) was performed by ELISA using a CK1α antibody. (**D**) Purified GST-CK1α splice variant 1 or 2 or GST alone were coated on the ELISA plate and a titration of purified untagged MDM2 was added in the mobile phase. Antibody against MDM2 and the appropriate secondary antibody were used to quantify the binding using ECL (relative light units (RLU)). (**E**) Purified GST-cleaved MDM2 or control buffer alone (“c sp1” and “c sp2”) were coated on the ELISA plate and a titration of purified GST-CK1α splice variant 1 or 2 added. Antibody against CK1α and the appropriate secondary antibody were used to quantify the binding using ECL (in relative light units (RLU)). The figure is representative of two independent experiments in which each data point was assessed in triplicate. (**F**) A375 cells were transfected with either empty vector (lane 1) or the One-STReP-tagged CK1α splice variants 1 (lane 2) and 2 (lane 3). After gentle lysis, the lysates were incubated with STReP-Tactin beads and bound proteins eluted by heating at 85°C in SDS/DTT sample buffer. Protein levels from the lysates (lanes 1–3) and the matching eluates (lanes 4–6) were analyzed by Western blotting with antibodies targeting the indicated proteins.

MDM2 has been shown previously to co-immunoprecipitate with the CK1α isoform [16]. In order to assess if this was true using different MDM2 antibodies, we performed co-immunoprecipitations with these antibodies from the same lysate. The MDM2-CK1α complex was isolated with each of the antibodies, notably the 2A10 monoclonal antibody (Figure 2A upper panel esp. lane 5) that also showed the greatest quantity of immunoprecipitated MDM2 (Figure 2A lower panel). In the same experiment (Figure S3A & B), even though CK1α was co-immunoprecipitated with MDM2 in A375 cells, CK1δ and ε failed to be detected even though they were previously shown to interact with MDM2 [45]. The endogenous MDM2-CK1α complex was also captured by immunoprecipitation using CK1α antibody (Figure 2B lane 15). With MDM2 immunoprecipitations, only the lower and also more abundant band of the two input bands observed using the CK1α antibody is clearly co-immunoprecipitated, which could possibly reflect the lack of sensitivity of the assay (Figure 2A esp. lane 5, Figure 2B lane 13, upper panels). This observation suggested that the dominant of the two potential “isoforms” of CK1α was interacting with MDM2 and led us to further investigate the nature of these two bands observed by CK1α immunoblotting in cell lysates (Figure 1A lanes 1 & 5; Figure 2B lane 11) in order to further refine the complexity of the various isoforms of CK1. Both bands appear to represent CK1α, since *CK1α* siRNA decreased each of them (Figure 1A lane 8 vs. 7 & 4 vs. 3).

**Figure 3:**
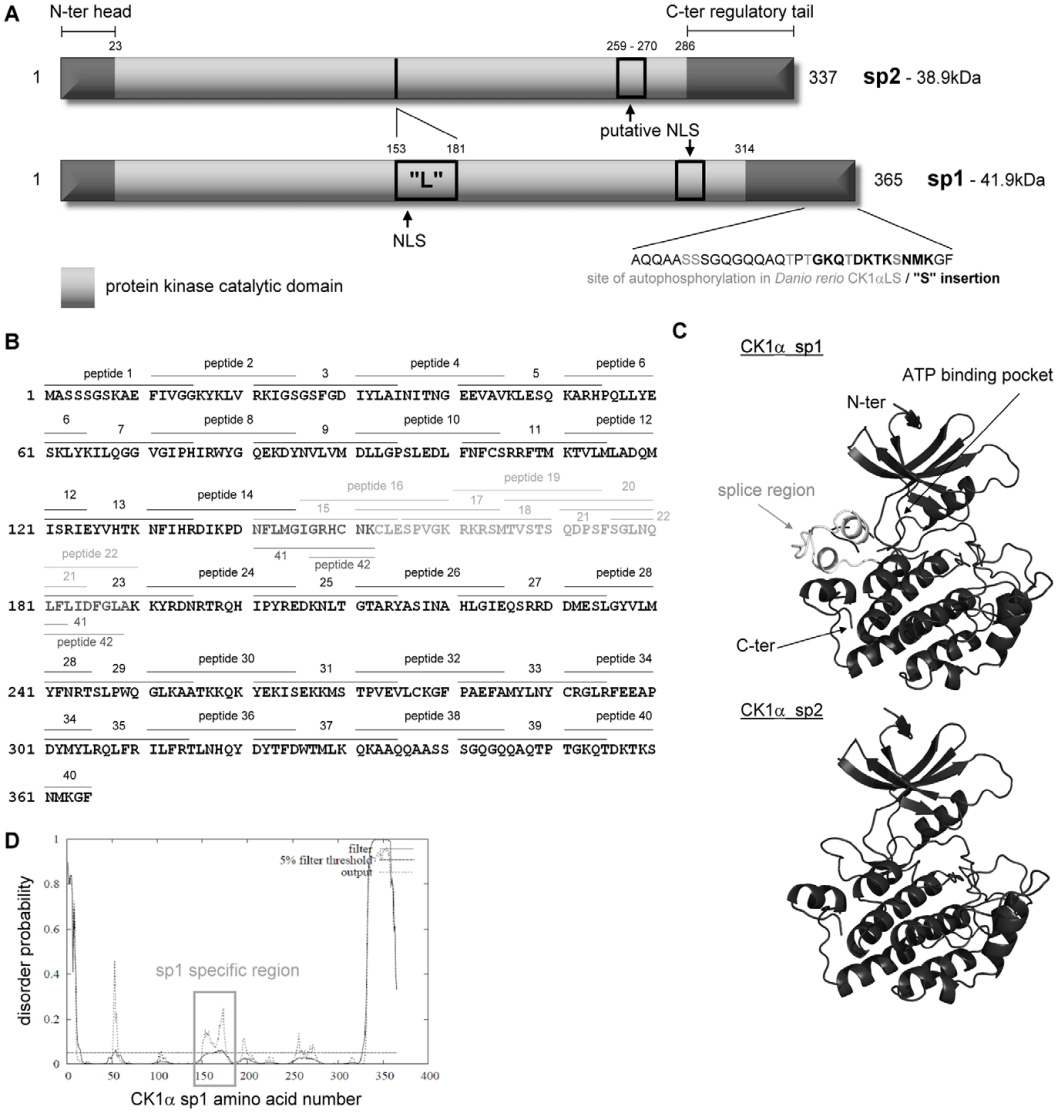
Features of CK1α transcript variants 1 and 2 and design of overlapping peptides. (**A**) Schematic representation of CK1α splice variant 1/2 highlighting the main features of CK1α including the nuclear localisation sequences (NLS) and the serine/threonine kinase catalytic domain. The “L” insertion is present in CK1α sp1 (aligning with CK1αLS from *Danio rerio*) but spliced in CK1α sp2 (CK1αS). The “S” insertion can be spliced in zebrafish, but the existence of a corresponding splice variant remains unknown in humans. (**B**) Overlapping peptides that were 15-amino acids long were designed spanning the entire length of the CK1α protein, featuring a 5-amino acid overlap at each extremity, with an N-terminal biotin tag and SGSG spacer. The light-grey amino acid sequences are specific to CK1α splice variant 1. Therefore peptides 15 to 22 with tighter overlap are splice variant 1 specific, and dark-grey peptides 41–42 are splice variant 2 specific. (**C**) Secondary structure cartoons of CK1α (amino acid 10-335 for sp1 and 10-307 for sp2) were generated using SWISS-MODEL which is an automated protein structure homology-modelling server. It predicted the three dimensional structures of CK1α sequences based on their similarities with the experimentally determined crystal structure of CK1δ protein (PDB ID: 1CKI_A). In white is shown the region where the alternatively spliced sequence of CK1α transcript variant 1 is inserted. N-, C-terminus and the position of the ATP-binding cleft are pointed out by arrows on the cartoon structure. (**D**) Disorder probability analysis of the CK1α protein sequence was determined using DISOPRED2 with a false positive rate of 5% (filter threshold). The filter curve corresponds to the output from DISOPRED2, and the output curve from DISOPREDsvm indicates shorter, low confidence predictions of disorder (http://bioinf.cs.ucl.ac.uk/disopred/).

From our available knowledge [8], [46], [47] on the *CK1α* gene in humans, there are two known transcript variants resulting from alternative splicing of *CSNK1A1.* They are CK1α transcript/splice variant 1 (or sp1; 365 amino acids) with a predicted size of 42 kDa that would correspond to the upper band, and CK1α sp2 (exon 5 is spliced out) with a size of 39 kDa matching the lower band (Figure 3A). To evaluate whether these two bands are likely to be CK1α sp1 and 2, both CK1α splice variants were cloned and expressed as untagged proteins using an *in vitro* transcription/translation kit (Figure S4 lanes 2&3 respectively). Indeed, the size of each *in vitro* translated CK1α matched one band of CK1α transcript variants from A375 lysate (Figure S4 lane 4).

Additionally, when MDM2 was transfected into A375 cells, the quantity of CK1α sp2 being co-immunoprecipitated with MDM2 didn’t increase even though the amount of MDM2 immunoprecipitated was considerably enhanced (Figure 2B lane 17 vs. 13). CK1α sp1 was however pulled down with increased level of exogenous MDM2. Curiously, the amount of MDM2 being co-immunoprecipitated with same level of CK1α was increased when MDM2 was transfected (Figure 2B lane 15 vs. 19), highlighting the possible presence of different cellular pools of MDM2-CK1α complex.

As a first step to test if there is an intrinsic difference in the binding between MDM2 and CK1α splice variants, ELISA experiments were performed using titrations of bacterially expressed and purified MDM2 and GST-tagged CK1α sp1 and 2 whose amounts were carefully normalised by ELISA (Figure 2C). Binding of MDM2 to CK1α sp1 and 2 in the solid-phase was observed implying a direct association between both proteins, but no striking difference was seen in the relative values (Figure 2D). However, in the inverse ELISA, CK1α sp2 displayed greater binding to MDM2 in the solid-phase compared to sp1 (Figure 2E). A similar trend was detected by pull-down of strep-tagged CK1α sp1 and 2 transfected into A375 cells; only sp2 exhibited elevated binding to endogenous MDM2 (Figure 2F lane 6 vs. 4&5). Therefore, since CK1α sp2 seems to be the major splice variant present in cells and in addition displays greater intrinsic binding to MDM2 *in vitro* as well as *in vivo*, subsequent experiments mainly used transcript variant 2.

### Fine-mapping the CK1α-MDM2 Protein Interface

The use of *Biologic* tools, such as aptamers, peptide mimetics or nanobodies provides novel approaches to dissect signalling pathways. We therefore aimed to develop peptides representing the MDM2-CK1α interface in order to uncover potentially novel concepts in cell growth control and to develop novel therapeutic leads for targeting this complex in cancer cells. In order to define the dominant contacts in the CK1α-MDM2 interface, a CK1α overlapping peptide library was synthesised and used in a series of competitive and direct binding experiments. These peptides were 15 amino acids long with a 5 amino acid overlap on each side, and featured an N-terminal biotin tag separated from the peptide sequence by an SGSG spacer (Figure 3B). Special care was taken in the design of the spliced region peptides which had a greater degree of overlap (peptides 15–22) and some additional peptides were designed to span the spliced region (peptides 41–42) that is present only in CK1α sp1 (Figure 3A). The spliced region does not appear in any other CK1 isoforms and since only the 3D structure of CK1δ but not α has been determined, an automated protein structure homology modelling server SWISS-Model was used to determine the most probable CK1α 3D structures [48], [49] (Figure 3C). This region is interesting as it appears to have a tendency for disorder (Figure 3D) but to a lesser extent than the CK1α N-terminal head and C-terminal regulatory tail (Figure 3A & D).

**Figure 4:**
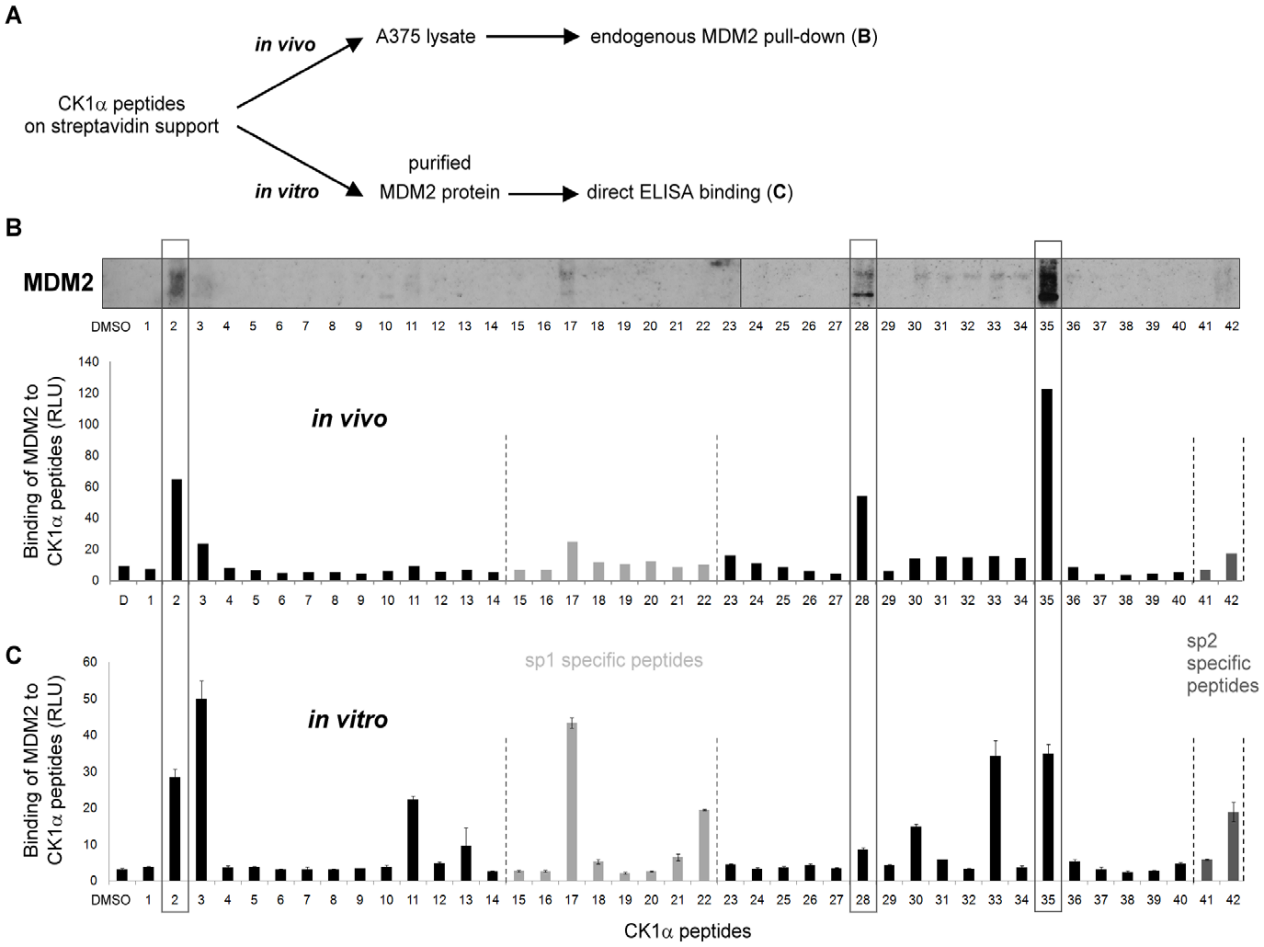
MDM2 binds to a dominant small motif within CK1α. (**A**) Biotinylated CK1α peptides were used to generate streptavidin-based peptide aptamer affinity columns for cell lysate MDM2 interaction assessment (**B**), or were coupled to streptavidin coated plates for direct ELISA binding assay (**C**). (**B**) Pre-cleared A375 lysate was applied to peptide aptamer affinity columns. A DMSO control was included. Eluates from the peptide aptamer affinity columns were analyzed on 10% gels and MDM2 binding was analyzed on immunoblots probed with anti-MDM2. Quantification of bound MDM2 protein level was performed using Scion Image software. (**C**) Mapping of MDM2 binding to overlapping CK1α peptides by ELISA. Overlapping CK1α peptides were coupled to streptavidin coated plates. Purified GST-cleaved MDM2 was added to each peptide and antibody against MDM2 and the appropriate secondary antibody were used to quantify the binding using ECL (in relative light units (RLU)). A DMSO control was included and values were normalised against the BSA background. The data are representative of three independent experiments in which each data point was assessed in triplicate.

*In vivo* pull-down experiments from A375 cell lysate were performed with each of the CK1α peptides immobilised on streptavidin-agarose beads (Figure 4A & B). Peptides 2, 28 and 35 captured the most endogenous MDM2 from the lysate. They differ in their abilities to associate with cellular MDM2, highlighting the specificity of the binding assay; peptide 35 captures MDM2 far better than peptides 2 and 28 (Figure 4B). In order to corroborate these results in an independent assay, recombinant MDM2 binding to CK1α peptides was evaluated by ELISA (Figure 4A & C) and revealed a similar pattern. A summary of MDM2 binding to CK1α peptides by either ELISA and *in vivo* pull-down is displayed in Table 1. Interestingly, peptide 2 which showed significant binding to MDM2 contains a potential p53 *BOX-I-*like motif. Interaction between the p53-*BOX-I* domain and the N-terminus/hydrophobic pocket of MDM2 was shown to promote conformational changes in MDM2 that stabilize acidic domain interactions with an ubiquitination signal in the DNA binding domain (*BOX-V*) of the p53 tetramer [31]. Therefore, peptides 2, 28 and 35 that bound specifically to cellular MDM2, as well as recombinant MDM2, were brought forward for MDM2-CK1α interface study.

**Table 1:** Summary of the significance of selected CK1α peptides in the linear binding peptide interface of CK1α-MDM2 complex.

CK1 peptide number	1	2	28	**35**
MDM2 binding to CK1peptides by ELISA	**–**	++	+	++
MDM2 from lysate bindingto CK1 peptide columns	**–**	+	+	++
Peptide competition ofCK1α-MDM2 binding	**–**	–	+	++
Peptide competition of p53ubiquitination by MDM2	**–**	++	+	+++
Score of peptide/9	0	5	4	**9**

### Key CK1α Peptides Disrupt the CK1α-MDM2 Complex and Inhibit MDM2’s E3 Ligase Activity

**Figure 5:**
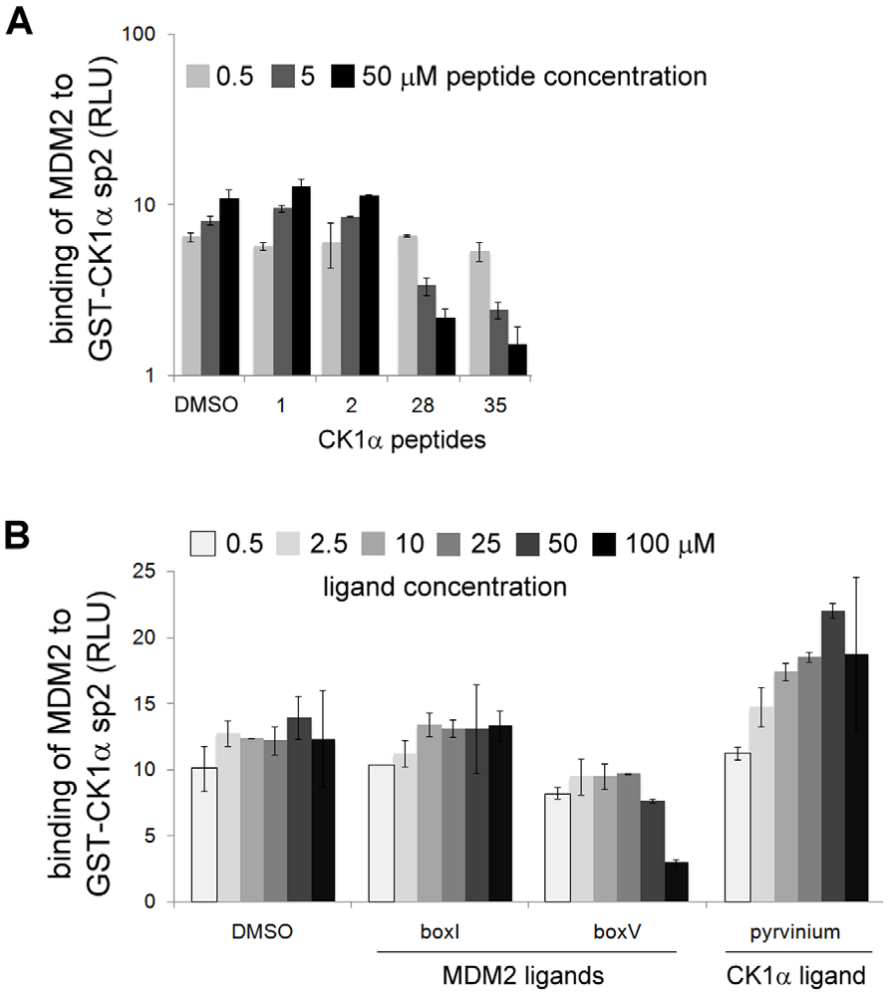
Key CK1α peptides can inhibit CK1α-MDM2 complex formation *in vitro.* GST-CK1α splice variant 2 was coated on ELISA plates. Purified GST-cleaved MDM2 was pre-incubated (with the exception of pyrvinium which was not pre-incubated but added to the plate at the same time as MDM2) with (**A**) CK1α peptides, namely peptides 1, 2, 28 or 35 (the data are representative of two independent experiments), or (**B**) MDM2 ligands (BOX-I and BOX-V) and CK1 stimulatory ligand pyrvinium, and then added to the plate. DMSO controls and a non-binding peptide (peptide 1) were included. MDM2 binding to CK1α was quantified using an antibody specific for MDM2 and an appropriate peroxidise-conjugated secondary antibody, by enhanced chemiluminescence (ECL) as relative light units (RLU). Each data point was assessed in triplicate.

To assess if the CK1α peptides which displayed binding affinity to MDM2 by ELISA and *in vivo* pull-down were able to disrupt the binding between CK1α and MDM2, peptide competition ELISAs were performed on the same basis as direct binding assays (Figure 2D) but with an additional peptide-MDM2 pre-incubation step. A titration of the panel of selected peptides was pre-incubated with MDM2 protein before addition to CK1α sp2 coated on the plate. Peptide 1 was used as a control since it did not bind to MDM2 in ELISA and pull-down experiments (Figure 4B & C). Only peptides 28 and 35, but not peptides 1 or 2, were able to decrease the binding of MDM2 to CK1α in a dose-dependent manner (Figure 5A). A quantitative summary of these results is displayed in Table 1. With the purpose of evaluating these data, binding between CK1α and MDM2 was assessed after pre-incubation with reference peptides known to bind different regions of MDM2: BOX-I, derived from the N-terminus of p53 which was shown to bind to the hydrophobic pocket of MDM2; BOX-V from the DNA binding domain of p53 that was shown to bind the acidic domain of MDM2 [31]. Pyrvinium was also included, which is an appealing molecule recently shown to bind selectively to CK1α and to allosterically activate CK1α *in vitro* [23]. The binding of the BOX-I peptide, like peptide 2, did not affect the CK1α-MDM2 complex at all (Figure 5B). On the other hand, BOX-V competed the binding in a similar manner to peptide 35 or 28 (Figure 5A & B). Lastly, pyrvinium increased the binding between CK1α and MDM2 (Figure 5B). Interestingly, pyrvinium was previously shown to selectively potentiate CK1α kinase activity [23] which highlighted the ability of pyrvinium as a small molecule tool for perhaps stimulating CK1α pathways.

**Figure 6:**
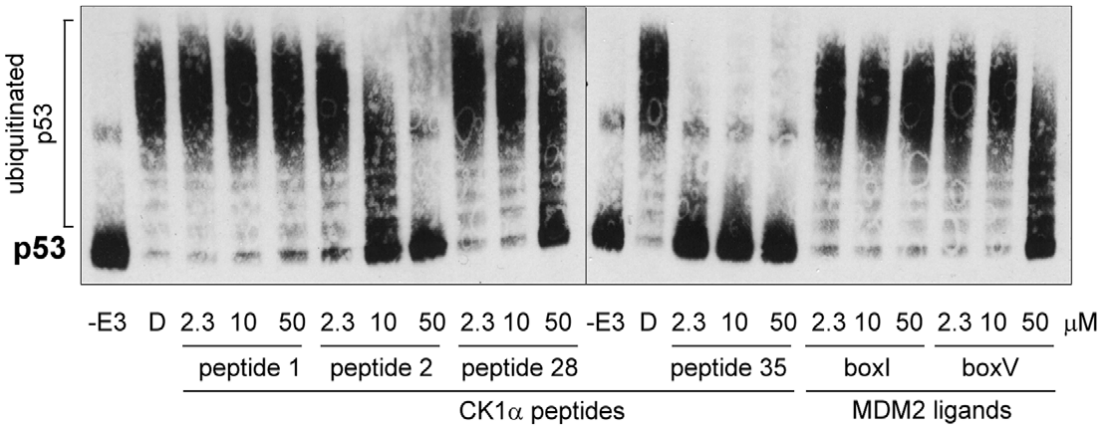
MDM2 interacting peptides derived from the sequence of CK1α can inhibit the E3 ubiquitin ligase function of MDM2 *in vitro.* In a peptide competition ubiquitination assay, untagged p53 protein purified from Sf9 insect cells was incubated with ubiquitin, UBE1 (E1), UbcH5a (E2) and ATP in the presence of full-length MDM2 pre-incubated with selected CK1α peptide 1, 2, 28 or 35, or BOX-I/−V) MDM2 ligands. Reactions were performed for 5 min at 37°C. Samples were analysed by immunoblotting with an anti-p53 antibody. A control with no MDM2 (“-E3”, peptide 2+ p53 only) and a DMSO control (“D”) were included.

Since two CK1α peptides were able to effectively block the CK1α-MDM2 interaction by binding to MDM2, we wanted to determine whether these peptides would also be able to inhibit MDM2’s E3 ubiquitin ligase activity. To perform this test, we used a well-established assay based on *in vitro* ubiquitination of p53 by MDM2 [31], [50]. Without MDM2, p53 appeared as a single band (Figure 6 lane “-E3”) which was replaced by an ubiquitinated adducts ladder in the presence of MDM2 (Figure 6 lane “D”). CK1α peptides that were brought to light in the previous CK1α-MDM2 binding assays, also had the ability to inhibit p53 ubiquitination by MDM2 in a similar manner as has previously been reported for BOX-V peptide [31]. Peptide 2 almost completely inhibited p53 ubiquitination at 50 µM and peptide 28 was also inhibitory but to a lesser extent, while peptide 35 inhibited MDM2 E3 ligase activity from 2.3 µM (Figure 6). The comparative significance of the effects of each peptide in the ubiquitination assay is summarized in Table 1.

**Figure 7:**
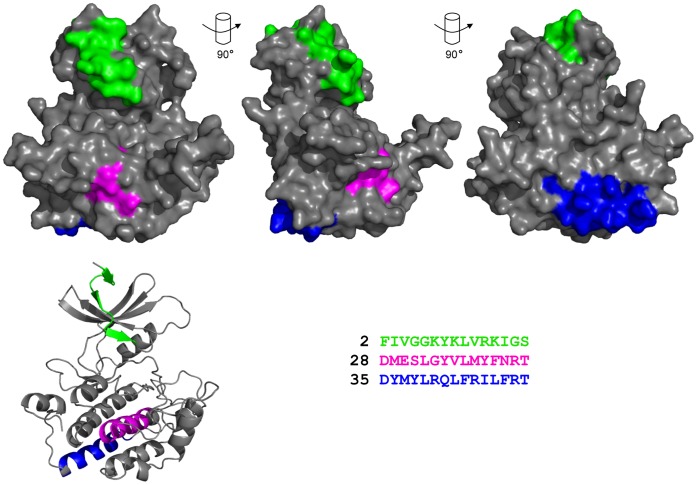
Analysis of MDM2 binding motifs within the overall three dimensional structural homology model of CK1α. Space-filling representation of CK1α sp2 generated using SWISS-MODEL (see figure 3 for details), is displayed at different angles. Secondary structure image of the primary space-filling molecule is shown in the bottom left corner. Regions highlighted in various colours correspond to CK1α peptides 2, 28 and 35.

Using the relative bioactivity of each peptide within the described assays assessing the CK1α-MDM2 complex (see Table 1), the significance of each peptide was assessed by giving an overall mark. Thus, peptide 35 is underlined as having the best score for being implicated in disrupting MDM2-CK1α binding. The peptide 35 motif is well exposed on the “bottom” surface of CK1 (Figure 7) and was chosen as the best candidate interface peptide between the MDM2 oncoprotein and the kinase. Peptide 2 is a prominence located just above the ATP-binding pocket of CK1 (Figure 7) and was excluded from the study since it was not bioactive in all four assays (i) ELISA with recombinant MDM2; (ii) cell lysate pull-down towards endogenous MDM2; (iii) MDM2-CK1α protein-protein interaction complex-based competition ELISA; and (iv) MDM2-mediated ubiquitination.

### The Bioactive Peptide 35 Triggers Changes in Cell Viability and a G0-G1 Growth Arrest in a p53 Independent Manner

**Figure 8:**
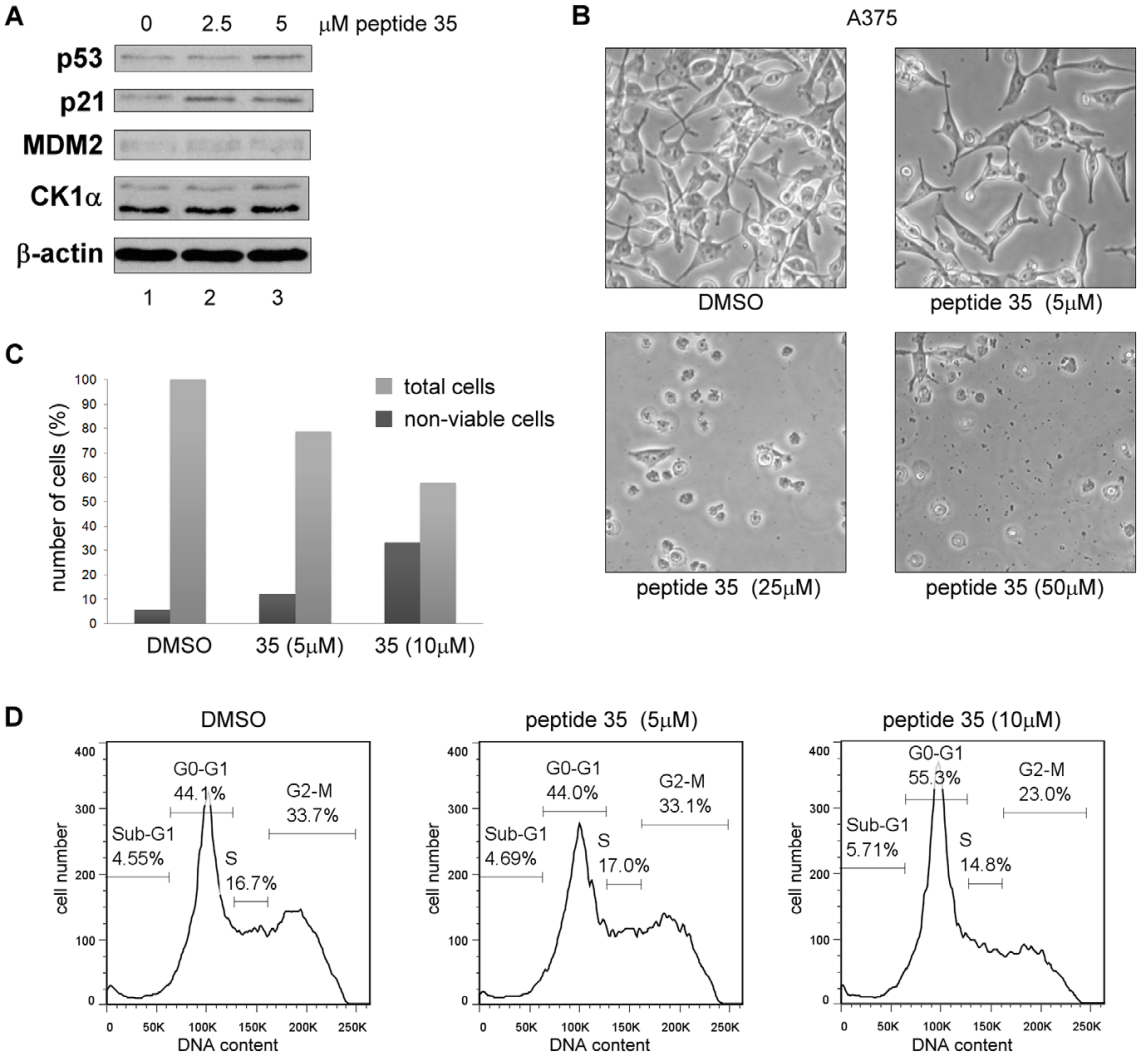
The CK1α peptide derived from its dominant MDM2 binding site triggers G0-G1 arrest and cell death in A375 cells. CK1α peptide 35 was transfected into A375 cells using Nucleofectin reagent and Nucleofector device II. A DMSO control was included. (**A**) Protein levels were assessed 18 hours after peptide transfection by Western blotting. (**B**) Images of cells were captured 16 hours after transfection. (**C**) The number of non-viable cells was counted after treatment using Trypan Blue. Total cells are shown in percent relative to the DMSO control and percent of non-viable cells are expressed relative to each respective total cell count. (**D**) After treatment, the cells were harvested then fixed in ethanol followed by staining with propidium iodide. DNA content was determined by FACS and analyzed with FlowJo7 software.

**Figure 9:**
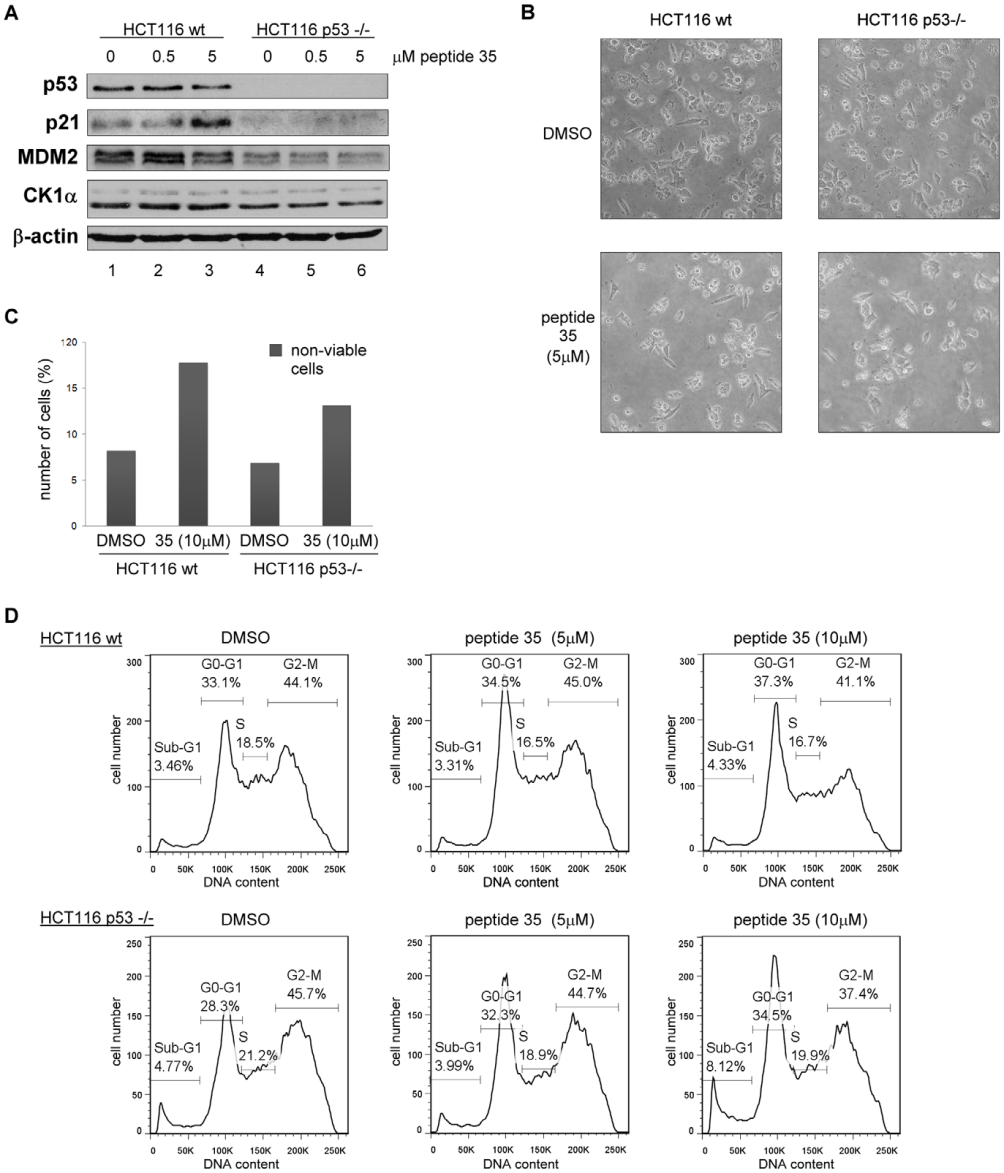
CK1α peptide derived from its dominant MDM2 binding site triggers G0-G1 arrest and cell death in a p53-independent manner. CK1α peptide 35 was transfected into HCT116 cells using Nucleofectin reagent and Nucleofector device II. A DMSO control was included. (**A**) Protein levels were assessed 18 hours after peptide transfection by Western blotting. (**B**) Images of cells were captured 16 hours after transfection. (**C**) The number of non-viable cells was counted after treatment using Trypan Blue. The percentage of non-viable cells is expressed relative to each respective total cell count. (**D**) After treatment, the cells were harvested then fixed in ethanol followed by staining with propidium iodide. DNA content was determined by FACS and analyzed with FlowJo7 software.

In a manner similar to CK1α siRNA and D4476, we investigated the possibility that CK1α peptides that competed with full-length CK1α for binding to MDM2 could also trigger the p53 pathway in A375 cancer cell line. To evaluate this hypothesis, the highly scoring peptide 35 (Table 1) was transfected into cells using Nucleofectin transfection reagent and electroporation. Using this approach, peptide 35 caused no striking increase in p53 levels (Figure 8A & 9A), but its downstream target p21 increased significantly compared to DMSO control (Figure 8A). In the same experiment, protein levels from cells transfected with higher concentrations of peptide 35 could not be measured accurately as cells were mostly all dead (Figure 8B). A lower titration of peptide 35 was therefore used and even at concentrations as low as 5 µM a growth inhibitory effect was seen on the cells, that were floating and non-viable at higher concentrations (Figure 8B). This low µM bioactivity of peptide 35 in cells is suggestive of a potent *Biologic* tool. Indeed increasing concentration of peptide 35 triggered a reduction of total cell number compared to the control alongside an increase in the proportion of non-viable cells (Figure 8C). In order to characterise this cell viability effect, FACS analysis was performed based on propidium iodide staining of the cell DNA, 18 hours after transfection of low levels of peptide 35. At a 10 µM peptide 35 concentration in A375 cells, a significant G0–G1 growth arrest was observed with an 11% increase compared to the DMSO control (Figure 8D).

Since p53 protein was seen to only increase slightly at higher peptide 35 concentrations, the possibility that the observed growth arrest would be p53-independent was assessed by using HCT116 wt and p53−/− cells (Figure 9). Indeed, the decrease in cell viability after peptide 35 transfection was observed with a similar intensity in both HCT116 cell lines (Figure 9B & C). FACS profiles also showed an increasing G0-G1 cell population in the presence of increasing peptide concentrations (Figure 9D). An additional 1.7-fold increase of apoptotic sub-G1 population was observed in p53−/− cells, which could be explained by previously suggested anti-apoptotic role of p53 [51]. No p53 increase but only a p21 protein level increase was observed after transfection of HCT116 wt (Figure 9A). Possibly linked to the absence of p53, no increase of p21 was observed in p53−/− cells, which would suggest that the observed G0-G1growth arrest was not linked to the p21 protein level increase (Figure 9A & D).

### Bioactive Peptide 35 Induces Ubiquitination and NEDDylation-like Modification of the Splice Variant 2 CK1α

**Figure 10:**
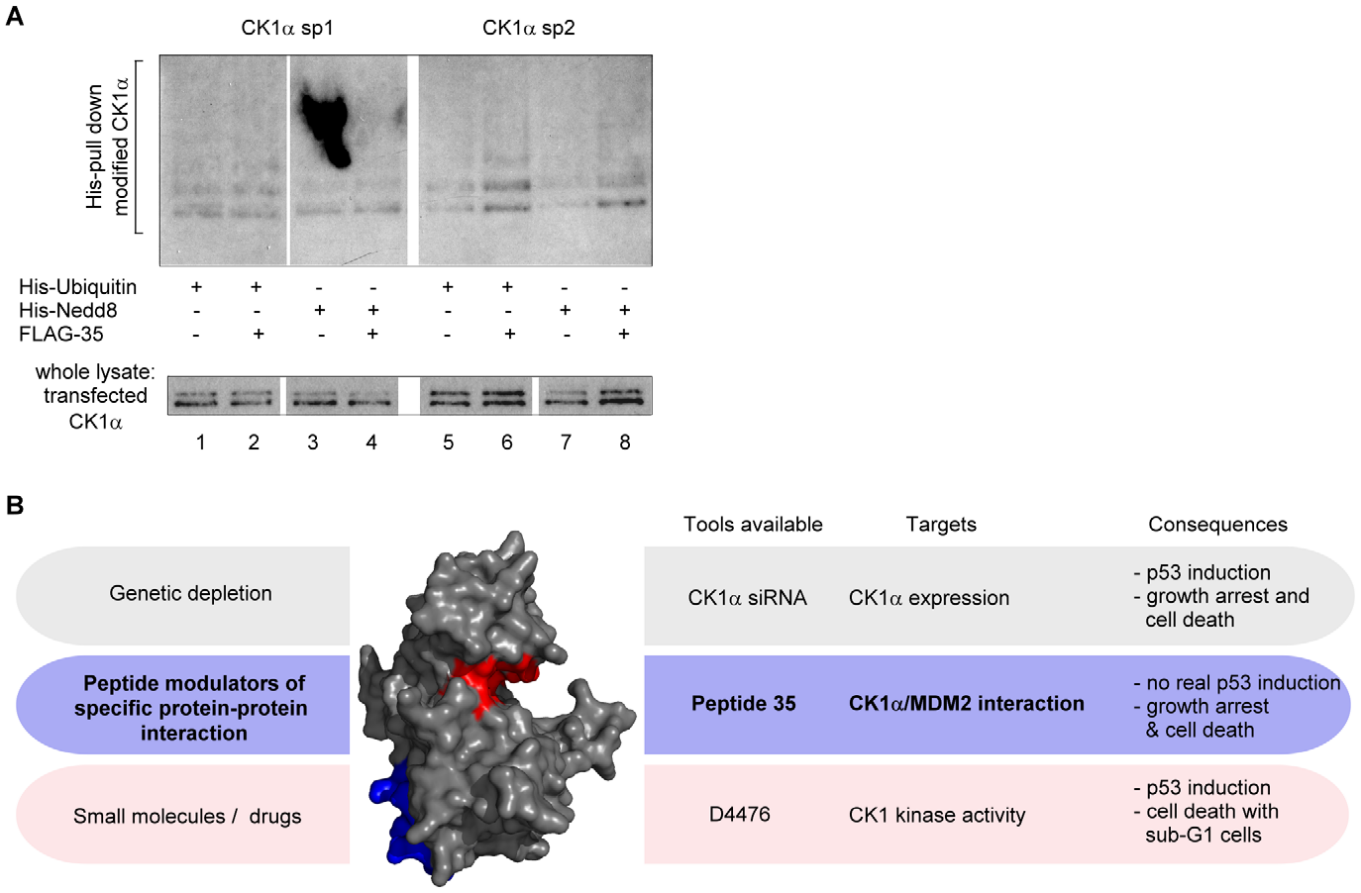
Outline of pharmacological manipulation of CK1 functions and its effects on p53 signalling pathway, cell viability, and CK1α post-translational modifications. (**A**) H1299 cells were transfected with STReP-tagged CK1α sp1 or 2, FLAG-tagged peptide 35 and His-Ubiquitin or His-Nedd8, using Attractene. Eighteen hours after transfection cells were treated with 50 µM MG132 for 4 hours. Whole cell lysates were analysed for transfected CK1α levels, and His-ubiquitin/NEDD8 conjugates were purified by metal affinity chromatography and analyzed by 4%–12% NuPAGE/immunoblots with an anti-CK1α antibody. Changes highlighted were representative of two independent experiments. (**B**) *Three approaches for manipulation of signal transduction pathways.* Genetic depletion of CK1α using siRNA or the CK1 kinase attenuation using the ATP-competitive inhibitor D4476 can alter the p53 response and cell viability, as indicated. The bioactive peptide from the high affinity MDM2-CK1α interface (peptide 35) highlights a novel peptide lead for disrupting signalling in cancers. All three approaches gave rise to overlapping but distinct effects on signalling. Specific siRNA against CK1α led to p53 induction and growth arrest accompanied with slight sub-G1 increase, whereas non-specific CK1 drugs such as D4476 mediated p53 induction and significant apoptosis. Low levels of the bioactive peptide 35 did not lead to p53 protein induction but to a growth arrest in G0-G1 and reduced cell viability. These data suggest that peptide 35 may function in a pharmacologically novel manner compared to the ATP-active site inhibitor or CK1α siRNA and highlights the general utility of targeting protein-protein interactions as approaches for developing therapeutic strategies that target signalling mechanisms in cancer.

Overall, peptide transfection did not trigger a significant p53 protein level increase although it induced noteworthy growth arrest and cell death from as little as 5 µM transfected-peptide concentrations, in a p53-independent manner (Figure 8 & 9 & 10B). Potentially novel interactions/modifications of CK1α *in vivo* after peptide 35 disruption of the CK1α-MDM2 complex might give new insights into cell death signalling events. To address this, we cloned peptide 35 in frame with an N-terminal FLAG tag. Ubiquitin and NEDDylation-like modifications were assessed after FLAG-tagged peptide 35 co-transfection with STrEP-tagged CK1α sp1 or 2 in H1299 cells. This experimental approach revealed significant increase of these post-translational modifications for CK1α splice variant 2 but not sp1 (Figure 10A), emphasizing novel interactions/modifications of CK1α following its release from the E3 ligase MDM2. These data highlight how an approach to develop a *Biologic* tool based on a protein-protein interface can give rise to novel information about cellular signalling; in this case we have identified a peptide that can induce growth arrest and reduce cell viability in a p53-independent manner, and co-ordinately induce ubiquitination and novel NEDDylation signals to CK1α that might explain in part alterations in growth responses. The further optimisation of peptide 35 as an MDM2-targeting lead that co-ordinates NEDDylation might provide a novel pharmacological tool to test as an anti-cancer therapeutic.

## Discussion

MDM2 is a known inhibitor of p53, but MDM2 also has over one-hundred other interacting proteins whose link to p53 inhibition and cell growth controls are not well defined; nor is there an annotation of which of these over hundred MDM2 interactors are dominant or less significant which would clarify their importance as therapeutic targets in cancer [27]. We have previously evaluated the CK1 family as established MDM2 interactors, to determine whether they are part of the same genetic p53-inhibitory pathway as MDM2. Previous published data indicated that blocking MDM2 with Nutlin-3a or depleting/inhibiting CK1α generated the same genetic effects: an increase of p53, MDM2 and p21 protein levels ([16], Figure 1A). Thus we propose that of the many MDM2 binding proteins, CK1α is a dominant MDM2 interacting protein. Sub-cellular fractionation experiments showed that CK1 inhibition induces significantly p53 nuclear levels thus allowing its transcriptional activity (Figure 1B). We previously proposed a molecular mechanism by which the p53-MDM2 association is regulated through the modulation of recruitment of protein kinases such as CK1, thus influencing CK1 substrate selectivity. The present study investigated the interaction mechanism between CK1α and MDM2 protein in order to further our understanding of the role of CK1α in regulating p53-MDM2 signal transduction pathways and to investigate whether novel domains in CK1α can be identified (forming *Biologics* tools) to stimulate further research (Figure 1C).

The genetic cooperation of CK1α and MDM2 as an “onco-complex” that inhibits p53 and has proliferating associated functions was tested by dissecting the protein-protein interaction interface between CK1α and MDM2. Two key interfaces are already identified in MDM2 that bind CK1: (i) phosphorylation sites in the acidic domain of MDM2 that activate it [38]; and (ii) a DSG motif (also known as phosphodegron) surrounded by numerous close potential phosphorylation sites, some of which could be phosphorylated by CK1 in a primed or non-primed manner [52]. Phosphorylation of this degron leads to recognition by β-TrCP E3 ligase, resulting in MDM2 degradation [45].

Mapping the interfaces between CK1α and MDM2 highlighted specific linear peptide motifs as binding signals or interfaces CK1α presented an N-terminal p53 *BOX-I*-like motif that is a canonical MDM2 hydrophobic pocket binding site. This is relatively novel in itself, as although a similar p53 dual-site docking ubiquitination mechanism by MDM2 was shown to operate on the IRF-2 transcription factor [53] and cell-fate determinant NUMB [54], only few proteins are known to possess a *BOX-I* motif and interact with MDM2. Another observation from the 3D structure of CK1 is that the first MDM2-binding motif is located within a loop/β-sheet/loop structure over the ATP-binding pocket (Figure 7).

The most remarkable interface is located close to the C-terminus of CK1α, at the edge of the kinase domain, with peptide 35 located in the “base” area of the 3D structure of the kinase (Figure 7). Peptide 35 sequence is quite conserved between CK1 isoforms [2]. We could hence infer that similarly to CK1 drugs, peptide 35 would not only affect CK1α pathways but possibly other CK1 isoform protein-protein interactions. However, although peptide 35 sequence is conserved between CK1 isoforms, the double mutation of two non-conserved residues within CK1ε/CK1α peptide 35 interface abolished CK1ε specific interaction with Dishevelled protein [55], highlighting that even within a conserved region, single non-conserved residues could still drive specific isoform interactions.

Interestingly, here peptide 35 has been shown to induce reduction of cell viability in a p53 independent manner. We ventured the hypothesis that freeing CK1α from its complex with MDM2 (using small molecules or *Biologics*) is the key event triggering growth arrest/cell death. While the role of CK1α as a regulator of MDM2/p53 activity has been previously shown, new insights into its regulation are of potential interest since CK1α it is involved in so many pathways such as TGF-β signalling, virus responses, and DNA-damage responses [56]. Indeed an increase in CK1α protein levels was noticed after peptide 35 transfection; moreover ubiquitin and NEDDylation-like post-translational modifications of CK1α splice variant 2 are significantly increased. This is the first time a kinase has been shown to be NEDDylated to the best of our knowledge. These ubiquitin-like modifications might be priming events of the tumour suppressor functions of CK1α, when this it is freed from MDM2 interaction.

Our studies in A375/HCT116 cells suggest that CK1α can be a proto-oncoprotein by having anti-apoptotic and proliferative effects as its inhibition or depletion leads to p53 induction and growth arrest or apoptosis [16]. The same conclusion was drawn in a study showing that CK1α is required for proper cell cycle progression in the fertilized mouse oocyte [57]. But in another study (using cell lines distinct from A375/HCT116 cells), the contrary is suggested: from nevus to metastatic melanoma CK1α protein levels are increasingly down-regulated and are associated with invasive tumour progression via alterations in β-catenin stability [58]. Similar conclusions were made in colorectal/intestine models using mice that showed extensive invasiveness following the loss of CK1α therefore suggesting it functions as a tumour suppressor [18].

Additionally, expression levels of both splice variants of CK1α and their ratios greatly fluctuate from one cancer cell line to another as it is for CK1δ protein levels (data not shown), which underlie the importance to study their transcriptional, translational and post-translational regulation that will give a clue into their different roles. Previous studies have shown differences in properties between these two transcript variants in terms of activity, localisation and stability [8], [9], [46], [47] but have not evaluated their distinct roles. The mechanism of substrate specificity is only beginning to be investigated between CK1 isoforms, for example between δ/ε and α [55]. In our hands there was a slight difference in the interaction between the two CK1α isoforms and MDM2, and as a result we focused only on the dominant CK1α isoform, sp2, which was also specifically modified by ubiquitination and neddylation after peptide 35 transfection.

To conclude, our identification of a bioactive peptide motif in the “base” of CK1α (peptide 35, Figure 7) as a key binding site for MDM2 protein highlights a region involved in protein-protein interactions on CK1α. The peptide derived from this region in CK1α that competes CK1α from its interactor MDM2 (Figure 10B), can induce growth arrest/death of the cells. Indeed, as the interest in CK1 as a potential therapeutic target is only now being strongly documented, the next challenge will be the generation of specific protein-protein interaction inhibitors such as peptide 35 that will form the basis for a growing *Biologics* toolbox that contrast with genetic depletion and active site-directed small molecule inhibitor approaches. Further research would define peptide 35 binding to MDM2, but also what implications ubiquitin and neddylation post-translational modifications have on CK1α functions and properties.

## Materials and Methods

### Cell Lines

All the experiments were performed with the A375 cell line (obtained from Professor P. Smith, University of Cardiff, UK; [59]), which is an adherent human amelanotic malignant melanoma cell line, with the H1299 cell line (from the ATCC American Type Culture Collection; distributor LGC standards, Middlesec, UK), which is an adherent lung carcinoma cell line, or with HCT116 wild type (wt) and p53−/− cell lines (obtained from Dr B Vogelstein, Johns-Hopkins, University, USA; [60]), which are adherent human colon carcinoma cell lines. Cell stocks were maintained in DMEM, RPMI 1640 or Mc Coy’s 5A respectively (Gibco, Invitrogen) supplemented with 10% foetal bovine serum (Autogen Bioclear), at 37°C and 10% CO_2_ for A375 cell line or 5% for H1299 and HCT116 cell lines.

### Transient Transfection of Small Interfering RNA (siRNA)

siRNA to *CK1α* gene was obtained from Dharmacon (siGENOME SMARTpool against Human *CSNK1A1* (NM_001892)). siCONTROL non-targeting siRNA Pool#2 was used as a control (siRNA control). HCT116 cells were transfected using DharmaFECT (Dharmacon) according to the manufacturer’s instructions. The final concentration of siRNA used was 40nM and cells were incubated for 48 to 96 hours. DharmaFECT (Mock) and DMEM only controls (untreated) were included.

### Cell Treatments and Fractionation

D4476 (Calbiochem) was transfected using Attractene (Qiagen) into A375 cells as recommended by the supplier. Cells were treated with 40 µM of D4476 (final concentration) for 72 hours. Mock transfected/DMSO solvent control was included. DMSO was purchased from Sigma. Subcellular fractions of A375 cells treated with D4476 were prepared with ProteoExtract kit (Calbiochem) according to the manufacturer’s instructions. Proteins from each fraction (10 µg, quantified by Bradford) were subjected to Western Blotting. A Coomassie blue gel was also run in parallel.

### Urea Lysis and Western Blotting

Cells were harvested and lysed in urea buffer as described previously [16]. Proteins (20 µg lysate, determined by Bradford) were prepared in 4 × SDS sample buffer (SB) containing 0.2 M dithiothreitol (SDS/DTT SB) and were finally resolved by denaturing polyacrylamide gel electrophoresis and Western blotting as previously described [16]. Primary antibodies are listed in Table 2 and were used with the appropriate horseradish peroxidise (HRP)-conjugated secondary antibody (rabbit anti-mouse and swine anti-rabbit, DAKO; donkey anti-goat, Santa Cruz Biotechnology).

**Table 2:** Different primary antibodies used for Western blotting, ELISA and co-immunoprecipitation.

Target protein	Clone	Primary antibody type	Supplier
β-actin	AC-15	Mouse monoclonal	Sigma (#A5441)
CK1α	C-19	Goat polyclonal	Santa Cruz Biotechnology (#SC-6477)
CK1δ	–	Rabbit polyclonal	Bethyl Laboratories (#A302-136A)
CK1ε	–	Mouse monoclonal	BD Transduction Laboratories (#610445)
MDM2†	2A10	Mouse monoclonal	From Borek Vojtesek, Brno, Czech Republic*
MDM2†	4B2	Mouse monoclonal	From Borek Vojtesek, Brno, Czech Republic*
MDM2	2A9	Mouse monoclonal	From Borek Vojtesek, Brno, Czech Republic*
MDM2	3G5	Mouse monoclonal	From Borek Vojtesek, Brno, Czech Republic*
MDM2	SMP14	Mouse monoclonal	From Borek Vojtesek, Brno, Czech Republic*
p21	EA10	Mouse monoclonal	Calbiochem (#OP64)
p53	DO1	Mouse monoclonal	From Borek Vojtesek, Brno, Czech Republic*
HP1α	15.19s2	Mouse monoclonal	Millipore (#05-689)
HSP70	C92F3A-5	Mouse monoclonal	Stressgen (#SPA-810)

* Dept. of Experimental Oncology, Masaryk Memorial Cancer Institute, Brno, Czech Republic.

† Unless specified otherwise, a mix of both antibodies was used for Western Blotting; 2A10 was used in ELISAs.

### *In vivo* Co-immunoprecipitation

A375 cells were harvested, lysed in co-immunoprecipitation (co-IP) buffer and immunoprecipitation was performed as described previously [16]. Briefly, lysates were precleared with Protein G Sepharose (GE Healthcare) and then incubated with the indicated MDM2 primary antibodies or CK1α antibody (Table 2). A no antibody negative control was included. Protein G Sepharose was then added to the pre-cleared samples. Supernatant (flow-through) was collected and beads were washed several times with co-IP buffer. After addition of SDS/DTT SB, samples were boiled and eluates were collected. Samples were analysed by Western Blotting. In additional experiments, 4 µg of pCMV wild type MDM2 [31] or equivalent empty plasmid were transfected 24 hours prior to harvesting the cells.

### Cloning and Expression of CK1α for *in vivo* Pull-down

*CK1α* transcript variant 2 was amplified by PCR from cDNA obtained by reverse transcription of QPCR Human Reference total RNA (Stratagene) using the following primers: GCACTTCGGGATCCCATGGCGAGTAGCAGCGGCTCC (forward) and GACGTATCGTCGATATCTTAGAAACCTTTCATGTTAC (reverse). *CK1α* transcript variant 1 was amplified by PCR with the above primers from pCMV6-AC-GFP *CK1α* splice variant 1 (Origene). CK1α ORFs were cloned in frame with an N-terminal One-STrEP-TAG into pEXPR-IBA-105 (IBA BioTAGnology) using *Bam*HI and *Eco*RV restriction enzymes. A375 cells were transfected with 4 µg of the corresponding plasmid DNA, using Attractene as described in the manufacturer’s handbook. An empty vector only control was included. Cells were lysed in 0.2% Triton lysis buffer (50 mM HEPES (pH 7.5), 0.2% (v/v) Triton X-100, 150 mM NaCl, 10 mM NaF, 2 mM DTT, 0.1 mM EDTA,1XcOmplete Mini protease inhibitor cocktail (Roche)) 24 hours post transfection. Three mg of lysate was added to 80 µL (50% slurry) strep-Tactin MacroPrep (IBA) pre-washed in buffer W (100 mM Tris (pH 8.0), 150 mM NaCl, 1 mM EDTA, 1 mM benzamidine) and incubated for 1 hour at 4°C with gentle rotation. The resin was pelleted by centrifuging at 1000 g for 5 min at 4°C, and then washed several times with buffer W. OneSTrEP-proteins were eluted by the addition of 80 µL of SDS/DTT SB at 85°C for 5 min. Lysates (40 µg) and eluates (20 µL) were resolved by Western blotting.

### Cloning and Expression of CK1α for *in vitro* Studies

*CK1α* transcript variant 2 and 1 cDNAs were amplified by PCR from pEXPR-IBA-105 described above, using the following primers: GCACTTCGCATATGCATGGCGAGTAGCAGCGGCTCC (forward) and GACGTATCGTCCTCGAGTTAGAAACCTTTCATGTTAC (reverse). CK1α ORFs were cloned in frame with an N-terminal glutathione S-transferase (GST) tag into pGEX-6P-1 (GE Healthcare) using *Nde*I and *Xho*I restriction enzymes. *BL21(DE3)* competent *E. coli* cells were transformed with the above plasmids and expression of CK1α transcript variants was induced at 30°C for 3 hours with 1 mM IPTG at an optical density of 0.5 at 600 nM. Afterwards, cells were pelleted at 6000 g for 20 min at 4°C and cell pellets were resuspended in lysis buffer (20 mM Tris (pH8), 150 mM NaCl, 0.1% (v/v) NP-40, 1 mg/mL lysozyme, 2 mM DTT, 1× protease inhibitor cocktail). Following incubation on ice, cells were snap-frozen then quickly thawed and this freeze-thaw cycle was repeated. Cells were then disrupted using SHM2 Homogeniser (Stuart) and finally samples were centrifuged at 16000 g for 15 min at 4°C. Supernatants were combined with 50% slurry Glutathione Sepharose 4B beads (Amersham GE) prewashed in buffer A (20 mM Tris (pH8), 150 mM NaCl, 0.1% (v/v) NP-40, 2 mM DTT, 2 mM benzamidine) and incubated for 1 hour at 4°C with gentle rotation. Suspensions were mounted on 5 mL disposable MoBiCol columns (MoBiTec) fitted with 35 µm pore-size lower filters and Luer-lock caps and emptied by gravity. After washes in buffer A, further washes were performed in buffer B (buffer A supplemented with 10 mM MgCl_2_ and 5 mM ATP) then in buffer A. Recombinant proteins were eluted by incubation in elution buffer (100 mM Tris (pH 8), 120 mM NaCl, 40 mM glutathione) for 30 min at 4°C with rotation. Finally, the buffer was exchanged using Zeba Desalt Spin columns (Pierce) according the manufacturer’s instructions into kinase buffer (50 mM Tris (pH 8), 150 mM NaCl, 0.5 mM EDTA, 0.02% Triton X-100, 2 mM DTT).

### Expression and Purification of MDM2

MDM2 was expressed as GST-tagged protein using pGEX-6P-1 and GST was then cleaved off using PreScission protease (GE Healthcare) as described previously [61].

### Direct Protein-binding ELISA

Proteins used for direct protein-binding assays were GST-cleaved MDM2 and GST-tagged recombinant CK1α transcript variants 1 and 2. ELISA 96-well plates (Costar) were coated with either 20 ng of CK1α or 50 ng of MDM2 in coating buffer (0.1 M NaHCO_3_ pH 8.6) by incubating overnight at 4°C. GST alone and/or coating buffer only controls were included. After washing with PBS-T (PBS supplemented with 0.1% Tween-20), non-specific binding was blocked by incubation in blocking buffer (3% BSA in 1 × PBS) for 1 hour at room temperature. A titration of MDM2 or CK1α respectively in blocking buffer was then added and the plate was incubated for 1hour at room temperature. Following further washes, the plate was incubated with primary antibody specific to MDM2 or CK1α (Table 2) for 1 hour at room temperature. After washing, the appropriate HRP-conjugated secondary antibody was added and the plate was incubated for 1 hour at room temperature. After final washing, binding was measured by enhanced chemiluminescence (ECL). The luminescence produced was immediately detected with a Fluoroskan Ascent FL luminometer and Ascent software version 2.4.1 (Labsystems). BSA background was subtracted to normalise values.

### Peptide Affinity Chromatography-based Pull-down

A375 untreated cells were lysed in 0.1% Triton lysis buffer. The technique has been described previously [62]. Lysate was pre-cleared using Sepharose CL 4B beads prewashed in buffer W for 1 hour at 4°C with gentle shaking. Beads were centrifuged at 500 g for 3 min at 4°C and supernatant was collected and assayed for protein concentration. In parallel, peptide affinity columns were prepared using MoBiCol column jackets: 100 µl of 50% slurry streptavidin-agarose beads (Invitrogen) was added to each empty column and washed with buffer W. Ten µg of each of the CK1α biotinylated peptides in PBS was added to the individual columns and incubated for 1 hour at room temperature on a rotating table. A DMSO control was included. After several washes with buffer W, 0.5 mg of pre-cleared lysate was added to the peptide column and incubated with the resin for 1 hour at room temperature on a rotating table. Following further washes with buffer W supplemented with 0.2% Triton X-100 (v/v) and then buffer W, the beads were transferred from the columns to microfuge tubes which were then centrifuged at 500 g for 3 min at room temperature. Bound proteins were eluted by boiling in 50 µl SDS/DTT SB (100 mM DTT) for 5 min at 85°C. Beads were pelleted by centrifuging at 500 g for 3 min at room temperature, and the eluates were analysed by Western blotting.

### Peptide-based ELISA

Fifteen-mer biotinylated CK1α peptides (5-mer overlap at each extremity, SGSG spacer) were purchased from Chiron Mimotopes and dissolved at 5 mg/mL in DMSO. In peptide binding assays, ELISA plates were adsorbed with streptavidin (AnaSpec) overnight and washed with PBS-T, then biotinylated peptides diluted in water (1 µg per well) were added for 1 hour followed by blocking with 3% BSA in 1× PBS. Twenty ng of MDM2 diluted in blocking buffer were then added to the plate and incubated for a further 1 hour. Blocking buffer and DMSO only controls were included. After further washes, wells were incubated with MDM2 primary antibody and then the appropriate horseradish peroxidase antibody (Table 2) for 1 hour each at room temperature followed by further washing and ECL addition and the luminescence produced was measured. BSA background was subtracted to normalise values.

### Competition ELISA

The assay was carried out in a similar manner to direct protein-binding ELISA except that a fixed amount of MDM2 was pre-incubated 20 min at room temperature with a titration of peptide/drug (except for CK1α ligand Pyrvinium) in ELISA buffer (25 mM Hepes (pH 7.5), 50 mM KCl, 10 mM MgCl_2_) before addition to GST-CK1α transcript variant 2 (20 ng/well) coated on the plate. The p53 BOX-I and BOX-V sequences are PPLSQETFSDLWKLLP and RNSFEVRVCACGRD respectively. The CK1α stimulator molecule Pyrvinium was obtained from Ascent Scientific. BSA background was subtracted to normalise values.

### Peptide Competition Ubiquitination Assay

The *in vitro* assay was carried out as previously described [31] except using untagged p53 purified from insect cell lines as substrate (kindly donated by Jennifer A. Fraser, purified as previously published [63]) and reactions were started with MDM2 (20 ng/µL) pre-incubated for 10 min at room temperature with CK1α peptides (50 µM). A DMSO control and a no MDM2 control (p53+ peptide 2 only) were included. Samples were then incubated at 30°C for 15 min and reactions were stopped by addition of SDS/DTT SB. p53 ubiquitination was analyzed immunochemically by running 4%–12% NuPAGE gels in a MOPS buffer system (Invitrogen) followed by Western blotting with an anti-p53 antibody.

### Cloning of CK1α for Expression in Reticulocyte Lysate

*CK1α* transcript variants 1 and 2 were amplified by PCR from pEXPR-IBA-105 containing *CK1α* (see above) using the following primers: GCACTTCGGGATCCGCCACCATGGCGAGTAGCAGC (forward) and GACGTATCGTCCTCGAGTTAGAAACCTTTCATGTTAC (reverse). CK1α ORFs were then cloned into pcDNA3.1+ (Invitrogen) using *Bam*HI and *Xho*I restriction enzymes. CK1α transcript variants were then expressed using the TNT T7 Quick coupled Transcription/Translation kit (Promega) according to the manufacturer’s instructions. Five µg of the reaction was analysed by Western blotting alongside 30 µg of untreated A375 lysate.

### Peptide Transfection into Cells

Selected CK1α peptide (0.5 to 50 µM) was transfected into A375 or HCT116 cells using Nucleofectin reagent and Nucleofector II device following the manufacturer’s protocol (Amaxa Cell Line Nucleofector Kit V (LONZA)). After 18 hours, cells were harvested, lysed and protein levels were assessed by Western blotting. A DMSO control and/or control peptide were included.

### Cloning and Expression of FLAG-tagged CK1α Peptides

The DNA sequence encoding peptide 35 with an N-terminal SGSG spacer (GAATTCTTCAGGATCAGGAGATTACATGTATCTGAGGCAGCTATTCCGCATTCTTTTCAGGACCTAAGGATCC for peptide 35) was ligated into the 5′ *Eco*RI and 3′ *Bam*H1 sites of p3xFLAG-*Myc*-CMV-26 resulting in the removal of the *Myc* C-terminal tag. Therefore the FLAG-*Myc* empty plasmid was used as a peptide control.

### *In vivo* Purification of His-tagged Ubiquitin/NEDD8 Conjugates

H1299 cells were transfected with equivalent amounts (0.5 µg) of STReP-tagged CK1α splice variant (sp) 1 or 2, FLAG-tagged peptide 35 and His-Ubiquitin or His-Nedd8, using Attractene as described in the manufacturer’s handbook. Eighteen hours after transfection cells were treated with 50 µM MG132 (Calbiochem) for 4 hours. They were washed with PBS and scraped in 1 ml of PBS. Twenty percent of cell suspension was lysed in urea buffer. His-ubiquitin/nedd conjugates were purified as previously described [64] and analyzed by 4%–12% NuPAGE/immunoblots with an anti-CK1α antibody.

### Cell Cycle Analysis and Cell Counting

Cells were harvested in the medium by scraping on ice. Cells were then fixed, stained with propidium iodide and analysed by FACS analysis as described before [16]. A cell aliquot was taken first to count non-viable cells stained with Trypan Blue (Sigma) at 0.2% final concentration.

## Supporting Information

Figure S1 **Effects of CK1α depletion on the stability of CK1δ and ε isoforms.** A375 cells were transfected with control siRNA (40 nM; lanes 3 & 7) or with CK1α-specific siRNA (40 nM; lanes 4 & 8) for 48 hours (lanes 1–4) or 72 hours (lanes 5–8). A Mock transfected control (lanes 2 & 6) and an untreated control (DMEM only; lanes 1 & 5) were included. Cell lysates were analysed by Western blotting with antibodies targeting the indicated proteins.
(DOCX)

Figure S2 **Effects of CK1 inhibition on p53 and CK1 isoforms subcellular protein levels.** A375 cells were transfected with 40 µM final concentration of the CK1 inhibitor D4476 for 72 hours. Cells were then fractionated into four subcellular compartments: cytosol, membranes and membrane organelles, nucleic proteins, and cytoskeletal components. Cell subcellular lysates were immunoblotted (Figure 1B) and quantification of different protein levels in each fraction was assessed with Scion Image software. To remove any loading or protein concentration measurement inaccuracies, normalisation was performed by quantifying total protein levels of each fraction on a Coomassie blue gel (B) or the Ponceau-stained blot, then applying the difference factor between each fraction type to the Western blot protein quantification. The experiment was repeated at least three times (after 48 and 72 hours with 20 and 40 µM of D4476). Average p53 protein levels in each fraction before and after treatment are displayed in subfigure (C) which also shows standard deviations.
(DOCX)

Figure S3 **Co-immunoprecipitation of CK1 isoforms with MDM2 in A375 cells.** (**A**) MDM2 was immunoprecipitated from A375 cell lysate using the 2A10 monoclonal antibody. Co-immunoprecipitation was performed and included no antibody control and no lysate control. The flow-through (FT) and the eluate (E) for both controls and sample were analysed by Western blotting with anti-CK1α, δ and ε antibodies. Immunoprecipitation of MDM2 protein was checked with a 1∶1 mix of 2A10 and 4B2 antibodies. (**B**) Quantification of co-immunoprecipited CK1α splice variant 2 protein levels was assessed with Scion Image software in three independent MDM2 co-immunoprecipitation experiments.
(DOCX)

Figure S4 **Detection of CK1α splice variants by CK1α antibody.** Untagged CK1α splice variants 1 and 2 were expressed with an *in vitro* translation kit (IVT, lanes 2 and 3 respectively) and loaded alongside A375 lysate (lane 4); then examined by Western blotting with an antibody against CK1α. An empty control (pcDNA3.1) for *in vitro* translation was included (lane 1). The three panels represent decreasing exposure time of the same immunoblot. There is a noteworthy difference in the amount of both splice variants in A375 cell lysate, CK1α sp2 being about five fold more abundant than CK1α sp1 (lane 4). In a similar manner, sp2 was twofold more abundant than sp1 through expression with the *in vitro* kit (lane 2 vs. 3, right panel). This observation may highlight a difference in stability between the two splice variants.
(DOCX)
