# Supplementary material for: Exploiting the MDM2-CK1α Protein-Protein Interface to Develop Novel Biologics That Induce UBL-Kinase-Modification and Inhibit Cell Growth
Source: PLoS One. 2012 Aug 20;7(8):e43391. doi: 10.1371/journal.pone.0043391 (PMC3423359; doi:10.1371/journal.pone.0043391)
Supplement: Figure S1 — Effects of CK1α depletion on the stability of CK1δ and ε isoforms. A375 cells were transfected with control siRNA (40 nM; lanes 3 & 7) or with CK1α-specific siRNA (40 nM; lanes 4 & 8) for 48 hours (lanes 1–4) or 72 hours (lanes 5–8). A Mock transfected control (lanes 2 & 6) and an untreated control (DMEM only; lanes 1 & 5) were included. Cell lysates were analysed by Western blotting with antibodies targeting the indicated proteins. (DOCX) [file pone.0043391.s001.docx]

**Supporting information: Figure S1**


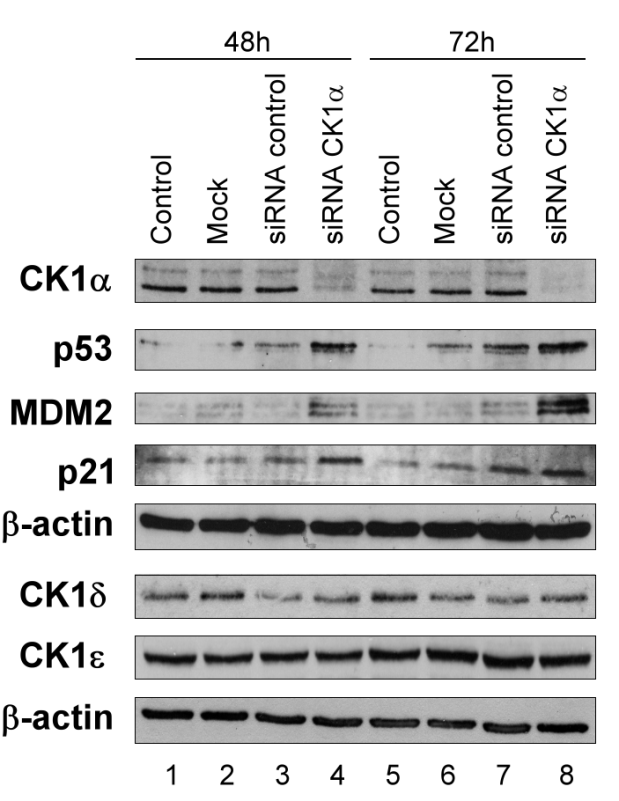


**Figure S1. Effects of CK1α depletion on the stability of CK1δ and ε isoforms.** A375 cells were transfected with control siRNA (40nM; lanes 3 & 7) or with CK1α-specific siRNA (40nM; lanes 4 & 8) for 48 hours (lanes 1-4) or 72 hours (lanes 5-8). A Mock transfected control (lanes 2 & 6) and an untreated control (DMEM only; lanes 1 & 5) were included. Cell lysates were analysed by Western blotting with antibodies targeting the indicated proteins.
