# Supplementary material for: Exploiting the MDM2-CK1α Protein-Protein Interface to Develop Novel Biologics That Induce UBL-Kinase-Modification and Inhibit Cell Growth
Source: PLoS One. 2012 Aug 20;7(8):e43391. doi: 10.1371/journal.pone.0043391 (PMC3423359; doi:10.1371/journal.pone.0043391)
Supplement: Figure S2 — Effects of CK1 inhibition on p53 and CK1 isoforms subcellular protein levels. A375 cells were transfected with 40 µM final concentration of the CK1 inhibitor D4476 for 72 hours. Cells were then fractionated into four subcellular compartments: cytosol, membranes and membrane organelles, nucleic proteins, and cytoskeletal components. Cell subcellular lysates were immunoblotted (Figure 1B) and quantification of different protein levels in each fraction was assessed with Scion Image software. To remove any loading or protein concentration measurement inaccuracies, normalisation was performed by quantifying total protein levels of each fraction on a Coomassie blue gel (B) or the Ponceau-stained blot, then applying the difference factor between each fraction type to the Western blot protein quantification. The experiment was repeated at least three times (after 48 and 72 hours with 20 and 40 µM of D4476). Average p53 protein levels in each fraction before and after treatment are displayed in subfigure (C) which also shows standard deviations. (DOCX) [file pone.0043391.s002.docx]

**Supporting information: Figure S2**


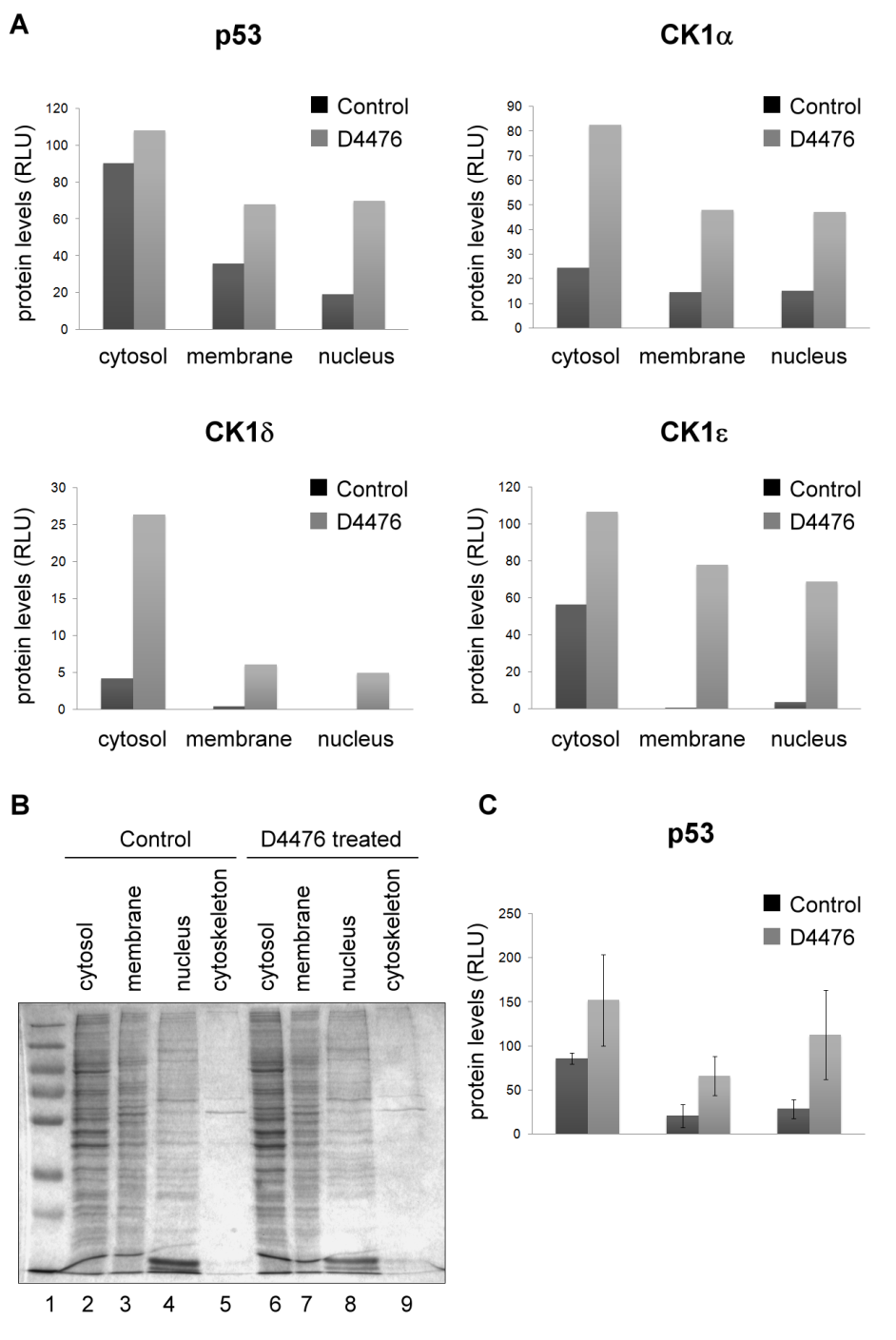


**Figure S2. Effects of CK1 inhibition on p53 and CK1 isoforms subcellular protein levels.** A375 cells were transfected with 40μM final concentration of the CK1 inhibitor D4476 for 72 hours. Cells were then fractionated into four subcellular compartments: cytosol, membranes and membrane organelles, nucleic proteins, and cytoskeletal components. Cell subcellular lysates were immunoblotted (Figure 1B) and quantification of different protein levels in each fraction was assessed with Scion Image software. To remove any loading or protein concentration measurement inaccuracies, normalisation was performed by quantifying total protein levels of each fraction on a Coomassie blue gel (B) or the Ponceau-stained blot, then applying the difference factor between each fraction type to the Western blot protein quantification. The experiment was repeated at least three times (after 48 and 72 hours with 20 and 40μM of D4476). Average p53 protein levels in each fraction before and after treatment are displayed in subfigure (C) which also shows standard deviations.
