# Supplementary material for: Exploiting the MDM2-CK1α Protein-Protein Interface to Develop Novel Biologics That Induce UBL-Kinase-Modification and Inhibit Cell Growth
Source: PLoS One. 2012 Aug 20;7(8):e43391. doi: 10.1371/journal.pone.0043391 (PMC3423359; doi:10.1371/journal.pone.0043391)
Supplement: Figure S3 — Co-immunoprecipitation of CK1 isoforms with MDM2 in A375 cells. (A) MDM2 was immunoprecipitated from A375 cell lysate using the 2A10 monoclonal antibody. Co-immunoprecipitation was performed and included no antibody control and no lysate control. The flow-through (FT) and the eluate (E) for both controls and sample were analysed by Western blotting with anti-CK1α, δ and ε antibodies. Immunoprecipitation of MDM2 protein was checked with a 1∶1 mix of 2A10 and 4B2 antibodies. (B) Quantification of co-immunoprecipited CK1α splice variant 2 protein levels was assessed with Scion Image software in three independent MDM2 co-immunoprecipitation experiments. (DOCX) [file pone.0043391.s003.docx]

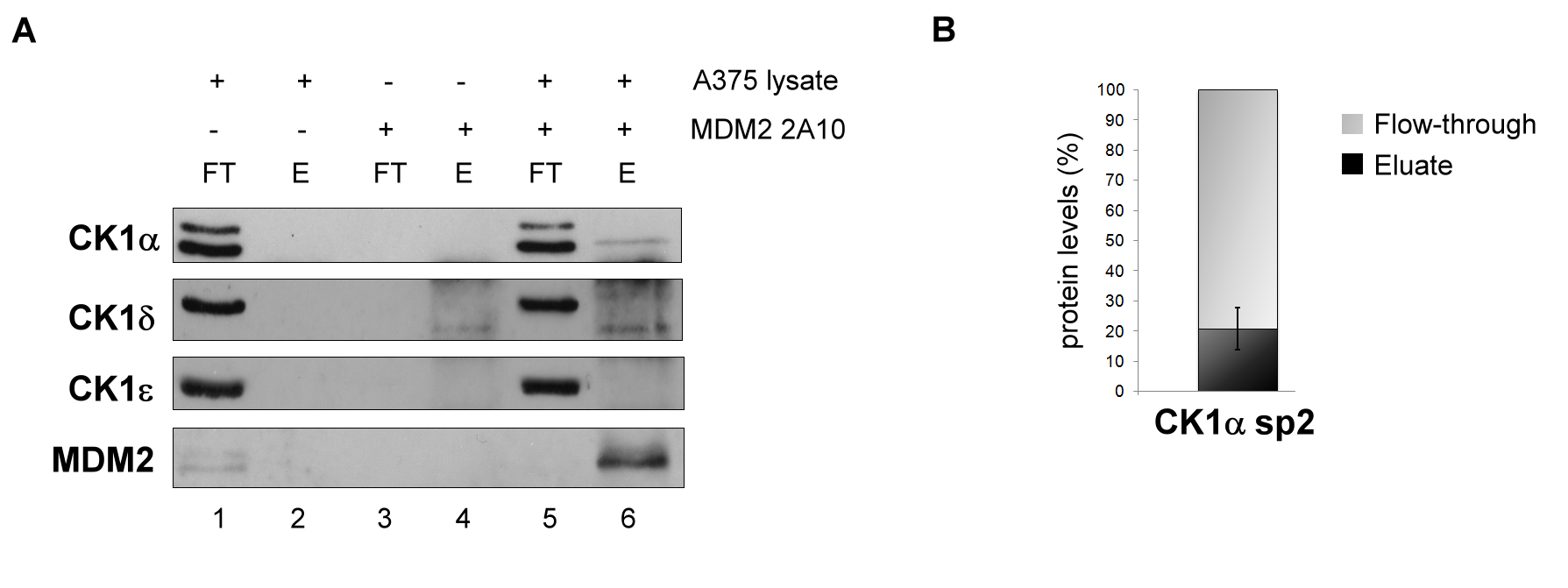


**Figure S3. Co-immunoprecipitation of CK1 isoforms with MDM2 in A375 cells.** (**A**) MDM2 was immunoprecipitated from A375 cell lysate using the 2A10 monoclonal antibody. Co-immunoprecipitation was performed and included no antibody control and no lysate control. The flow-through (FT) and the eluate (E) for both controls and sample were analysed by Western blotting with anti-CK1α, δ and ε antibodies. Immunoprecipitation of MDM2 protein was checked with a 1:1 mix of 2A10 and 4B2 antibodies. (**B**) Quantification of co-immunoprecipited CK1α splice variant 2 protein levels was assessed with Scion Image software in three independent MDM2 co-immunoprecipitation experiments.
