# Supplementary material for: Exploiting the MDM2-CK1α Protein-Protein Interface to Develop Novel Biologics That Induce UBL-Kinase-Modification and Inhibit Cell Growth
Source: PLoS One. 2012 Aug 20;7(8):e43391. doi: 10.1371/journal.pone.0043391 (PMC3423359; doi:10.1371/journal.pone.0043391)
Supplement: Figure S4 — Detection of CK1α splice variants by CK1α antibody. Untagged CK1α splice variants 1 and 2 were expressed with an in vitro translation kit (IVT, lanes 2 and 3 respectively) and loaded alongside A375 lysate (lane 4); then examined by Western blotting with an antibody against CK1α. An empty control (pcDNA3.1) for in vitro translation was included (lane 1). The three panels represent decreasing exposure time of the same immunoblot. There is a noteworthy difference in the amount of both splice variants in A375 cell lysate, CK1α sp2 being about five fold more abundant than CK1α sp1 (lane 4). In a similar manner, sp2 was twofold more abundant than sp1 through expression with the in vitro kit (lane 2 vs. 3, right panel). This observation may highlight a difference in stability between the two splice variants. (DOCX) [file pone.0043391.s004.docx]

**Supporting information: Figure S4**


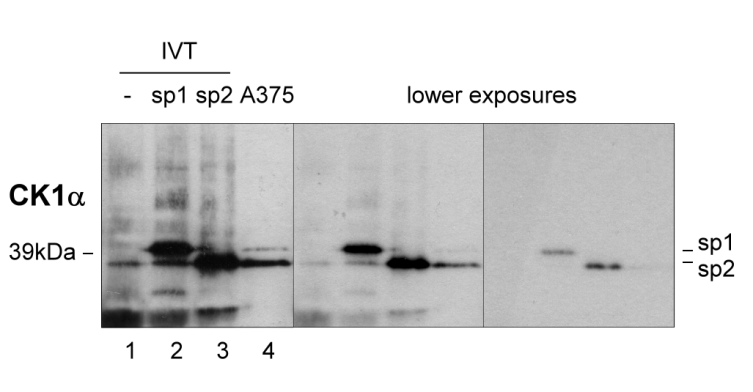


**Figure S4. Detection of CK1α splice variants by CK1α antibody.**  Untagged CK1α splice variants 1 and 2 were expressed with an *in vitro* translation kit (IVT, lanes 2 and 3 respectively) and loaded alongside A375 lysate (lane 4); then examined by Western blotting with an antibody against CK1α. An empty control (pcDNA3.1) for *in vitro* translation was included (lane 1). The three panels represent decreasing exposure time of the same immunoblot. There is a noteworthy difference in the amount of both splice variants in A375 cell lysate, CK1α sp2 being about five fold more abundant than CK1α sp1 (lane 4). In a similar manner, sp2 was two fold more abundant than sp1 through expression with the *in vitro* kit (lane 2 vs. 3, right panel). This observation may highlight a difference in stability between the two splice variants.
